# Mobilization of HIV Spread by Diaphanous 2 Dependent Filopodia in Infected Dendritic Cells

**DOI:** 10.1371/journal.ppat.1002762

**Published:** 2012-06-07

**Authors:** Anupriya Aggarwal, Tina L. Iemma, Ivy Shih, Timothy P. Newsome, Samantha McAllery, Anthony L. Cunningham, Stuart G. Turville

**Affiliations:** 1 Laboratory of HIV Biology, Immunovirology and Pathogenesis Program, The Kirby Institute, University of New South Wales, Sydney, New South Wales, Australia; 2 HIV Pathogenesis Laboratory, Westmead Millennium Institute (WMI), University of Sydney, Sydney, New South Wales, Australia; 3 School of Molecular Biosciences, University of Sydney, Sydney, New South Wales, Australia; Fred Hutchinson Cancer Research Center, United States of America

## Abstract

Paramount to the success of persistent viral infection is the ability of viruses to navigate hostile environments *en route* to future targets. In response to such obstacles, many viruses have developed the ability of establishing actin rich-membrane bridges to aid in future infections. Herein through dynamic imaging of HIV infected dendritic cells, we have observed how viral high-jacking of the actin/membrane network facilitates one of the most efficient forms of HIV spread. Within infected DC, viral egress is coupled to viral filopodia formation, with more than 90% of filopodia bearing immature HIV on their tips at extensions of 10 to 20 µm. Live imaging showed HIV filopodia routinely pivoting at their base, and projecting HIV virions at µm.sec^−1^ along repetitive arc trajectories. HIV filopodial dynamics lead to up to 800 DC to CD4 T cell contacts per hour, with selection of T cells culminating in multiple filopodia tethering and converging to envelope the CD4 T-cell membrane with budding HIV particles. Long viral filopodial formation was dependent on the formin diaphanous 2 (Diaph2), and not a dominant Arp2/3 filopodial pathway often associated with pathogenic actin polymerization. Manipulation of HIV Nef reduced HIV transfer 25-fold by reducing viral filopodia frequency, supporting the potency of DC HIV transfer was dependent on viral filopodia abundance. Thus our observations show HIV corrupts DC to CD4 T cell interactions by physically embedding at the leading edge contacts of long DC filopodial networks.

## Author Summary

Dendritic cells represent a unique cell type with respect to HIV, as they are the first point of contact for the virus in the genital mucosa and have the ability to spread virus efficiently in very low numbers to the primary HIV target, CD4 T cells. During the primary immune response, dendritic cells work in small numbers to make numerous and repetitive contacts, in order to filter and communicate with appropriate CD4 T cells. Thus HIV is hypothesized to be hijacking the same DC-CD4 T cell communication. Attempts to observe how HIV would achieve this have largely been limited, as introduction of imaging markers in the virus has often led to significant viral attenuation. Herein by using novel HIV constructs that permit imaging of HIV in infected dendritic cells, we observed newly forming HIV virions on the tips of long finger-like projections known as filopodia. In real-time imaging filopodia pivoted at their base and moved virions along trajectories that led to numerous CD4 T cell contacts. By manipulating filopodial formation we conclude the location of the virus on long filopodial tips allows the virus to corrupt the promiscuous dendritic cell to CD4 T cell contacts for efficient viral spread.

## Introduction

For HIV infections to persist, efficient spread to the next permissive target is paramount. HIV as a cell free entity is readily a target to both innate and acquired immune defenses, and by random diffusion alone, it must travel distances up to a thousand fold its diameter to make contact with a potential target. Even after this journey, permissive cells can be often equipped with various antiretroviral restriction factors, which would simply result in one of many dead ends for the virus. The above, described concepts are the underlying reasons cell to cell contact via a molecular structure termed a viral synapse plays a major role in maintaining viral persistence [1], [2], [3]. Viral synapses not only deliver the virus directly to the target, but also at high multiplicity, ensuring greater probability of a productive infection. In the context of cell-cell transmission, infected DC have long represented one of the most potent forms of cell associated HIV inocula for CD4 T cells [4], [5], [6], [7] and it is hypothesized that HIV subverts the normal immunological communication pathways between DC and CD4 T cells for viral broadcasting. The physiological importance of DC- HIV transfer is a function of their sentinel activities at the genital mucosa. This sentinel activity places DC as the first line of cells that come into contact with the virus and thus attention has been focused for some time on how DC can disseminate HIV infection to the major targets, CD4 T cells. Given DCs physiological location and the potent ability with which infected DC transfer virus, our main aim was to determine the mechanistic prerequisites of viral transfer between infected DC and CD4 T cells. Our focus on the *de novo* HIV pool in infected DC must not be confused with the extensive literature of DC HIV infection *in trans* [4], [8], [9], [10]. The *in trans* phenotype is defined as the binding/uptake of virus from the surrounding inoculum, which can be then transferred from DC to CD4 -T cells in the short-term (effectively between 4 to 6 hours) independent of DC infection [4], [8], [11], [12]. In contrast the *de novo* viral pool/phenotype, we define as the expression of viral proteins within an infected cell type that leads to particle assembly and transfer. The importance of the latter *de novo* pool in infected DC, as opposed to the viral pool in exposed immature DCs (*in trans* pool), is reasoned three-fold. Firstly, transfer from infected DCs from the *de novo* pool is greater in efficiency and duration than *in trans* pool in immature DC [4], [11], [12]. Secondly, the majority of DC subsets *in vivo*, in particular cutaneous and mucosal DC [7], [13], have been observed to mobilize viral transfer from the *de novo pool*, whereas the survival of HIV within the *in trans* pool is variable and in most cases limiting [4], [6], [7], [12], [14], [15], [16]. Finally, whilst recent studies do not support high frequencies of DC infection, due to the direct consequence of DC expressing the HIV restriction factor SAMHD1 [17], the majority of data to date observe only limiting numbers of infected DCs being readily capable of rapidly seeding CD4 T cell infections [5], [6], [7], [15]. Thus as limiting numbers of DCs can communicate the immune response, limiting numbers of infected DCs are equally capable of communicating HIV through CD4 T cell populations.

Whilst close viral synapses have been observed to be key to HIV transfer and infection, little is known what leads to their formation. For instance, an isolated infected cell needs to find a future permissive target, otherwise viral transfer through a synapse would never proceed. Recently HIV has been observed on filopodial like structures, termed viral cytonemes [18] and HIV T cell nanotubes [19]. Whilst both structures result in HIV transfer, their formation are dependent on previous cell-cell contacts and thus may not be not perceived as coordinating structures prior to cell-cell contact. In looking at potential mechanisms that may occur prior to cell-cell viral transfer, we initially hypothesized that co-ordination of closer and/or tethered cell-cell contacts proceeds via an actin based pathway. The support for an actin-based initiation of viral spread is in the mechanistic studies on many other other pathogens, including the bacteria Listeria, Shigella, Rickettsia and Burkolderia and viruses of the pox-virus family, where each pathogen has been observed to independently evolved to manipulate actin polymerization to aid in its own spread through the formation of filopodial-like structures termed actin tails (reviewed by [20]).

Herein, by focusing on the potent and important *de novo* HIV reservoir in infected DCs, we observe for the first time HIV utilizing cortical actin by embedding at the tips of networks of long myeloid enriched filopodia. Budding HIV and not mature HIV particles are observed on the ends of largely freely moving untethered filopodia and thus are not structures consistent with HIV cytonemes/nanotubes. As HIV caps the end of viral filopodia (VF), initially these novel structures were supported to be similar in nature to pathogenic actin tails. However, unlike actins tails, we observed VF formation to be not dependent on the Arp2/3 pathway but rather relying on expression on the formin Diaphanous 2 (Diaph2) for formation. In addition the accumulative evidence is VF are pre-existing filopodial networks that are high-jacked at the leading edge by newly forming virions, as opposed to pathogenic actin tails which are actively formed by the respective pathogen. The dynamic nature of the newly described VF, imparts three important advantages for the virus. Firstly, the virus can be positioned as close as possible to the target membrane and limit exposure of the newly forming virion to innate or acquired immune defenses. Secondly, the inherent probing and fast µm.sec^−1^ scanning motion of VF permit the virus to engage in a multitude of potential target contacts in a manner analogous to immune repertoire scanning by professional antigen presenting cell *in vivo*. Finally, as the virus is at the terminal stages of budding, it is physically tethered but primed for subsequent plasma membrane fission to be released to infect a CD4 T cell bound to an infected DC.

## Results

### Detection of the de novo pool in live DC reveals viral filopodia

**Figure 1:**
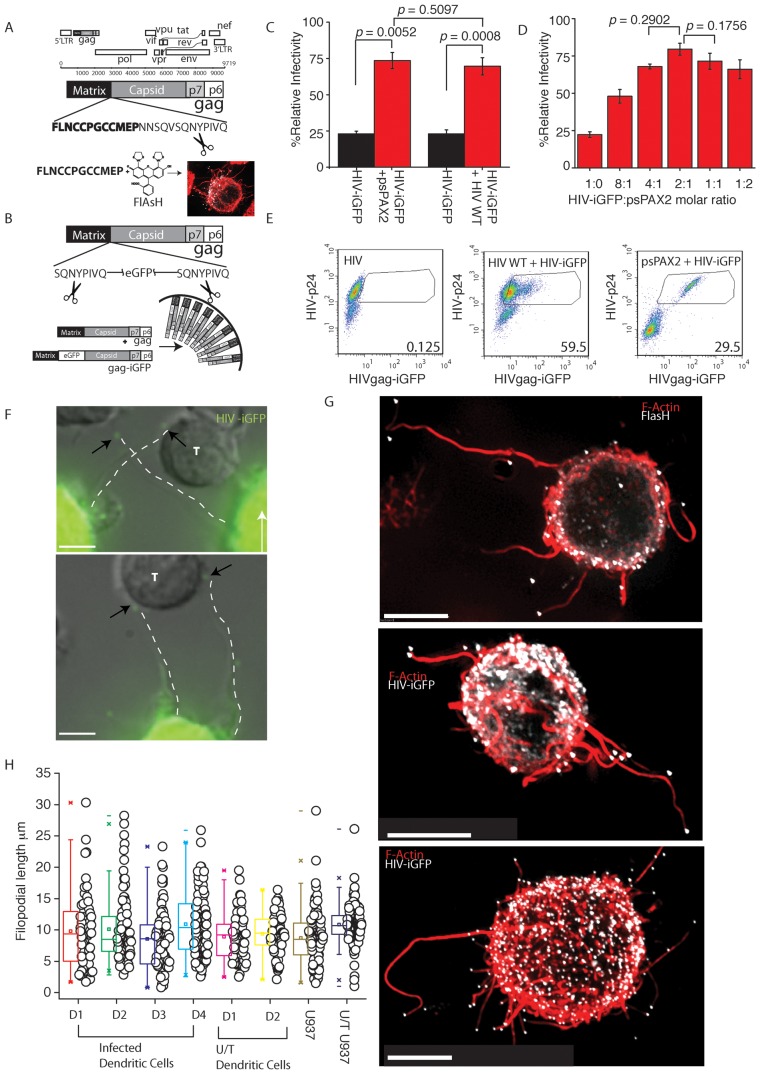
Complementary live imaging approaches reveal abundant HIV tipped filopodia (VF) on infected DC. (**A**) Detection strategy of HIV-T. The HIV Gag polyprotein is presented in the context of the HIV open reading frames. The biarsenical fluorescent dye FlAsH is shown and binds to a 12 amino acid motif (in bold) at the C-Terminus of Matrix. The protease cleavage site between HIV Matrix and Capsid is highlighted by black scissors. (**B**) Detection strategy using HIV-iGFP. HIV-iGFP constructs encode eGFP at the C-terminus of HIV matrix and are flanked by 5′ and 3′ HIV protease cleavage sites (highlighted by black scissors). For generation of cell-free virus with comparable infectivity to WT HIV Gag and Gag-Pol are expressed in trans to the HIV iGFP genome (see bottom of panel; HIV Gag only shown) to increase viral infectivity in one round. (**C**) **&** (**D**) Rescuing infectivity of HIV-iGFP. (**C**) HIV iGFP was prepared by co-transfecting WT HIV or WT Gag and Gag -POL (psPAX2) with HIV iGFP at an equimolar ratio into the 293T cell line. Three days post transfection, supernatants were harvested, diluted 1/1000 and 200 ml was added to 1×10^3^ TZM-bl cells (HeLa HIV indicator cell line), seeded 24 hours prior in a 96 well plate. % infectivity is relative to wild type HIV and calculated as the (co-transfections)/(HIV-WT alone)×100. Statistical differences are presented as *p* values. Standard deviations are derived from assays in triplicate. (**D**) Further titration of psPAX2∶HIV-iGFP. As in C. HIV-iGFP was co-transfected with psPAX2, but here at as a titration. Supernatants were subsequently titered using the TZM-bl cell line as in C. Standard deviations and *p* values also as per C. % infectivity relative to wild type HIV is calculated as in C. (**E**) DC were infected with an MOI of 0.1 with either WT HIV (left panel), WT HIV rescued HIV-iGFP virus (middle panel) or psPAX2 rescued HIV-iGFP (right panel) as outlined in material and methods. To determine total infection, infected DC were stained with anti-HIV-p24 antibody KC57-RD1. Gates in panels reflect eGFP signal from infected p24 high cells, with percentages from gates presented in the lower right corners. Note WT rescued HIV iGFP virus generates infected DC with diluted eGFP signal. (**F**) Infected DCs expressing HIV-iGFP 4 days post infection and co-cultured with autologous resting CD4 T cells at a ratio of 1 DC to 3 CD4 T cells (images are also representative for HIV-T). Filopodia are highlighted by dotted lines. Neighboring CD4 T cells that are in contact with filopodia are marked as (T) (**G**) HIV iGFP infected cells (HIV in white) have been fixed and stained for F-actin using phalloidin dye (red). Note all filopodia stained red, bear HIV at their terminal tips (All scale bars are 5 µm). (**H**) Average lengths of filopodia & VF across multiple DC donors. Infected or uninfected DCs (untreated U/T) were co-cultured with CD4 T cells as in F. Length of filopodia from the base at the plasma membrane to the tip was calculated in live infected and uninfected DC donors. VF and Filopodia lengths in infected and uninfected co-cultures from D1 & D2 are presented as a comparison. Filopodial lengths are representative of greater than *n* = 20 donors. VF and filopodia from infected and uninfected U937 cell line are also presented as a comparison.

To investigate the dynamics of HIV transfer from infected DC to CD4 T cells, we attempted to map this process in real-time. To do so we needed to undertake imaging of the *de novo* viral pool present in infected primary DC. To date imaging of the *de novo* pool has remained limited as markers amenable to imaging need to be integrated within the size sensitive HIV genome. The generation of HIV constructs by others and ourselves have addressed this short-coming by using small genetic tags that work in conjunction with cell permeable fluorescent dyes (termed hereon as HIV-T, Fig. 1A) [21], [22], or by overcoming the complications that large fluorescent proteins create when inserted into HIV structural proteins (termed HIV-iGFP, Fig. 1B) [23]. To persist with the use of traditional fluorescent proteins, we needed to rescue viral infectivity at two levels. Firstly we flanked the large fluorescent proteins with HIV protease cleavage sites (Fig. 1B) as previously described [23], thus giving the virus the ability to untether from the fluorescent protein when maturing. Secondly, we rescued this virus further by supplying wild type (WT) Gag and Gag-Pol *in trans* (Fig. 1C & D). Initially attempts of supplying WT Gag and Gag-Pol by co-transfecting WT HIV with HIV-iGFP did increase infectivity to levels comparable to WT HIV (Fig. 1C). However subsequent analysis of infected DC populations, highlight the dominance of the WT genome within the *de novo* pool (Fig. 1E HIV+HIV-iGFP). Thus to avoid a secondary WT competing genome in future *de novo* virus, we supplied WT HIV Gag and Gag-Pol *in trans* only at the protein level using the 2^nd^ generation lentiviral vector psPAX2 (Fig. 1D & E). When using this latter approach, we could not only rescue HIV-iGFP (Fig. 1C & D), but also readily infect primary DC (Fig. 1E; HIV+psPAX2 & Fig. S1A). More importantly, as there was no competing WT HIV genome, we could detect every cell that was infected by high levels of Gag-iGFP expression.

Using live microscopy, infected DC in CD4 T cell co-cultures were identified either by the presence of FlAsH staining for HIV-T or high eGFP fluorescence for HIV-iGFP. In both cases, punctate staining, presumably representing viral particles, was readily observed in the periphery of the DC plasma membrane and neighboring CD4 T cells (Fig. 1F). As these viral particles were highly motile, we further investigated whether they were still connected to the DC membrane. Peripheral viral particles remained connected by thin and often curved membrane extensions of the DC plasma membrane (Fig. 1F) and often provided bridge-like connections to neighboring CD4 T cells. Subsequent staining of fixed samples for filamentous actin using fluorescent phalloidin revealed HIV particles at the tips of F-actin rich filopodial like structures of variable length and curvature (Fig. 1G & H) and we now define these novel structures as HIV viral filopodia (VF). In live imaging of infected DC, the majority of VF (92.5% (*n* = 267 (D = 4)) were terminally capped with HIV Gag as shown by focal accumulation of eGFP and/or FlAsH at the filopodial tip (Fig. 1G. Video S1). Initially there were concerns that viral filopodial structures maybe a common HIV budding defect that was a result of HIV Gag fusion proteins both in HIV iGFP and HIV-T. To exclude this possibility, we determined the ability of HIV iGFP, HIV T and HIV iGFP+HIV WT (inocula identical to that use in Fig. 1E middle panel) infected DCs to transfer to autologous primary CD4 T cells. Virus transfer from HIV iGFP infected DCs was 1/3 that of wild type HIV infected DCs (Fig. S1B), although there there was no significant attenuation of HIV transfer from HIV T and HIV iGFP+HIV WT infected DCs to CD4 T cells compared to HIV WT infected DCs (Fig. S1B). As VF are also present in DCs infected with the latter viruses, the lack of viral attenuation in these important controls does not support the concept that VF are the product of a budding defect. Rather the attenuation that is observed in HIV iGFP is consistent with that of HIV viral entry, as cell free virus that is terminally mature [23] is also approximately 1/3 less infectious than HIV WT (Fig. 1C).

VF were variable in length from 1 µm to as long as 32 µm (10.11±5.42 µm, Fig. 1H, *n* = 267 (D = 5)). Given the complexity of DC membranes in the absence of infection, it was important to observe the frequency and phenotype of filopodia on uninfected DCs. Live DC cultures without CD4 T cells had polarized lamellipodia opposing a leading edge uropod (Video S2 with lamellipodia on the left of the uninfected DC). The latter structure expressed short filopodia (3.59+/−0.69 µm, *n* = 102) and were significantly shorter to VF (p<0.0001). However, as VF were imaged in the context of CD4-T cell co-cultures, we repeated filopodial analyses after DC that were co-cultured with autologous CD4 T cells for 30 minutes. The addition of CD4 T cells triggered filopodial formation to equivalent lengths of VF (Fig. 1H; Video S3; *p* = 0.387, *n* = 170). Thus the above observations initially supports HIV hijacking of existing filopodial networks, triggered in the presence of CD4 T cells. To determine whether filopodia could be capped on other cell types, we infected primary CD4 T cells. VF were expressed on primary activated HIV infected CD4 T cells, yet the VF frequency per cell was significantly lower compared to infected DC (1.26±0.72 VF.cell^−1^, *n* = 59 (D = 4) versus 8.89±5.17 VF.cell^−1^, *n* = 49 (D = 4)). In addition the average length of VF on CD4 T cell was shorter than on infected DC (4.69±2.124 µm, *n* = 138 (D = 4); *p*<0.0001). Uninfected T cells expressed similar filopodia, yet we could not identify co-culture conditions that triggered long filopodia analogous to uninfected DCs co-cultured with CD4 T cells. We then sought to identify a cell line that could express VF at similar lengths and frequencies to infected DC, thus permitting the use of experimental designs that may be limited in primary cells. Two decades ago, Phillips and colleagues observed a structure resembling VF using transmission electron microscopy in HIV chronically infected U937 cells engaging a neighboring epithelial cell [24], [25], we thus investigated whether the U937 cell line was expressing equivalent VF to infected DC. Indeed, we observed VF of equivalent length (8.71±3.99, *n* = 149, *p* = 0.206) and frequency per cell (13.27±4.59, *n* = 78, versus 8.89±5.17 VF.cell^−1^ *n* = 49 (D = 4)). As observed in infected DC, VF on U937s were terminally capped with HIV and consistent with that previously observed by Phillips and colleagues, yet in the later case was in the context of polarized filopodia engaged at a synaptic junction. Whilst VF on the U937 cell line were unpolarised, as was the case in infected DCs, we did observe filopodial polarization consistent with the image by Phillips *et al* (Video S4). Whilst we support VF on U937 and DCs to be similar, it must be noted U937 constitutively express long filopodia in both infected and uninfected U937 cultures (Fig. 1H) in the absence of CD4 T cell co-culture (unfortunately co-culture with autologous CD4 T cells was obviously not possible for this cell line).

### HIV viral filopodia trajectories repetitively scan at high velocities prior to CD4 T cell engagement followed by tethering

**Figure 2:**
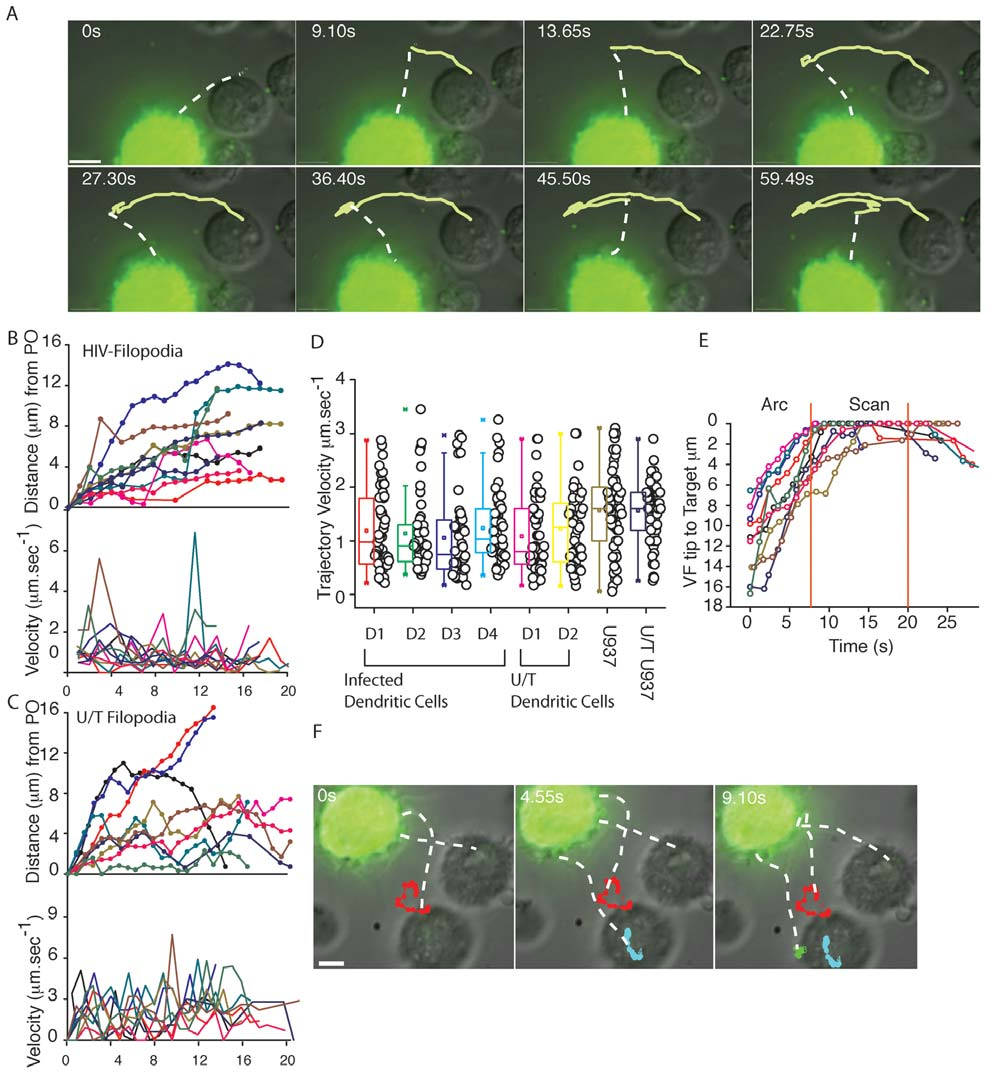
VF trajectories engage in fast overlapping Arc trajectories prior to engaging and scanning CD4 T cells. DC were infected with HIV-iGFP and subsequent live imaging proceeded with infected DCs co-culture with CD4 T cells at a ratio of 1 DC to 3 CD4 T cells as outlined in Fig. 1. (**A**) Untethered VF engage in sweeping Arc trajectories. To illustrate the overall movement of VF, 8 frames of the Supplementary Video S5 were superimposed. To highlight filopodia, a dashed line shadows the filopodial connection on VF as in Fig. 1. (**B**) 10 representative velocities of VF tips over time (lower graph) and corresponding movements from their point of origin (PO) (upper graph) (**C**) 10 representative velocities of filopodia from uninfected DC from the same donor. (**D**) Average velocities across entire VF trajectories across multiple DC donors and filopodial trajectories from untreated (U/T) controls (each point is the average VF Velocity over an entire 20 second trajectory). VF and filopodia from infected and untreated (U/T) U937 monocyte cell line is presented herein as a comparison. (**E**) Change in VF trajectories from Arc to Scan movements, when contacting CD4 T cells. The distance of the VF tip to the target T cells was calculated over time for 10 representative VF. Vertical red lines highlight the average scanning time of filopodia on the CD4 T cell membrane. Although scanning by normal filopdoia occurs, we could not resolve definitive trajectories as we did not have a tip marker equivalent to HIV on VF. (**F**) To illustrate the appearance of Scan trajectories in close contact with CD4 T cells, 3 frames have been taken from a live imaging time lapse experiment in Video S5, and single particle tracking over time highlighted in each frame. All scale bars are at 5 µm. All data is representative of in excess of 12 independent donors.

From hereon we use HIV iGFP as the primary tool in live imaging, as they are significantly brighter and photo bleach tolerant, which are key criteria for lower exposure times for fast acquisition of events during live imaging and the recording of longer trajectories for single particle analysis. Also at this point it is important to clarify the images in live cell acquisition versus the higher resolution images in fixed samples. In live cell acquisition there is readily detectable diffuse eGFP staining throughout the cell body, with low-level expression of eGFP at the filopodial tips. In contrast, fixed cell imaging there is resolution of HIV particles across the plasma membrane and at filopodial tips. This discrepancy is reasoned two-fold. Firstly, higher resolution fixed imaging is through the acquisition of entire infected cell volumes and subsequent 3- dimensional deconvolution [26]. In contrast in live cell imaging there was only acquisition of one Z-plane over time, thus lower resolution images due to the lack of entire Z-stack acquisition and subsequent deconvolution. The acquisition of only one Z-plane was a factor of VF dynamics, as acquisition of trajectories at velocities in excess of 1 µm.sec^−1^ limited the time needed for acquisition of a significant Z-stack. Secondly, the majority of fluorescence is within the cell body and was obviously limited at filopodial tips due to the relative small size of the virion. Thus exposure times that allow detection of virions on VF results in image acquisition that appear to overexpose the fluorescence in the cell body. Whilst the abovementioned fixed imaging conditions can remove the majority of out of focus light at the cell body, the restricted conditions of live imaging cannot. That said even with lower resolution images in live cell imaging, HIV particle detection at filopodial tips can be readily achieved, as the signal is of sufficient distance from the cell body to allow resolution. As we could readily resolve HIV particles on filopodia, we next characterized VF velocities and trajectories and the capacity of VF to be involved in HIV spread. We infected DCs and four days post infection we co-cultured them with autologous CD4 T cells. VF, when DC were not in immediate contact with CD4 T cells, displayed trajectories in arc-like movements, termed arc velocities, which included movements towards or away from CD4 T cells. Live acquisition of uninfected DCs observed similar arc trajectories, but only when DCs were co-cultured with CD4 T cells (Video S3). The trajectory of Arc velocities followed a single sweep, where the Arc velocity would slow or stop at the end of the trajectory and then often reversed and took the same path (Fig. 2A; Video S5). Analysis of velocity of both VF and uninfected DC filopodia observed trajectories with both acceleration to speeds >6 µm.sec^−1^ and deceleration with brief interludes of stationary pauses (Fig. 2B & C) with an average of 1.11±0.75 µm.sec^−1^ for VF (Fig. 2D; *n* = 124 trajectories (D = 4)) and 1.156±0.75 µm.sec^−1^ for uninfected DC filopodia (Fig. 2D; *n* = 113 trajectories (D = 4); *p* = 0.766 versus VF). VF Arc velocities terminated once in contact with the CD4 T cell membrane (Fig. 2E–F; Video S6 & S7). At that time, we have observed a movement significantly slower used by VF to scan CD4 membrane surface that we have termed Scan velocity (Fig. 2D–E; *p*<0.0001 for Arc versus Scan velocities; *n* = 52 (D = 4); Video S7). VF Scan velocity was 0.3422±0.196 µm.sec^−1^ for an average duration of 13.717±10.577 seconds (*n* = 52) (D = 4)). Unfortunately the tips of filopodia expressed on uninfected DCs could not be resolved when in the vicinity of CD4 T cells (given they lacked an equivalent tip marker to HIV on VF) and thus the equivalent dynamics of CD4 T cell contact could not be observed. To determine if VF Arc and Scan trajectories were simply a function of random Brownian motion, we calculated their respective Hurst Exponent as previously described [27], [28]. The Hurst Exponent (H) mathematically classifies trajectories as random (H = 0.5), directional (H>0.5), or confined movements (H<0.5). Whereas Arc velocities had limited variation in H and were significantly directional (H = 0.740±0.134; *p*<0.0001; *n* = 52), scan velocities ranged from confined to directional movements (H = 0.428±0.234; *n* = 52).

**Figure 3:**
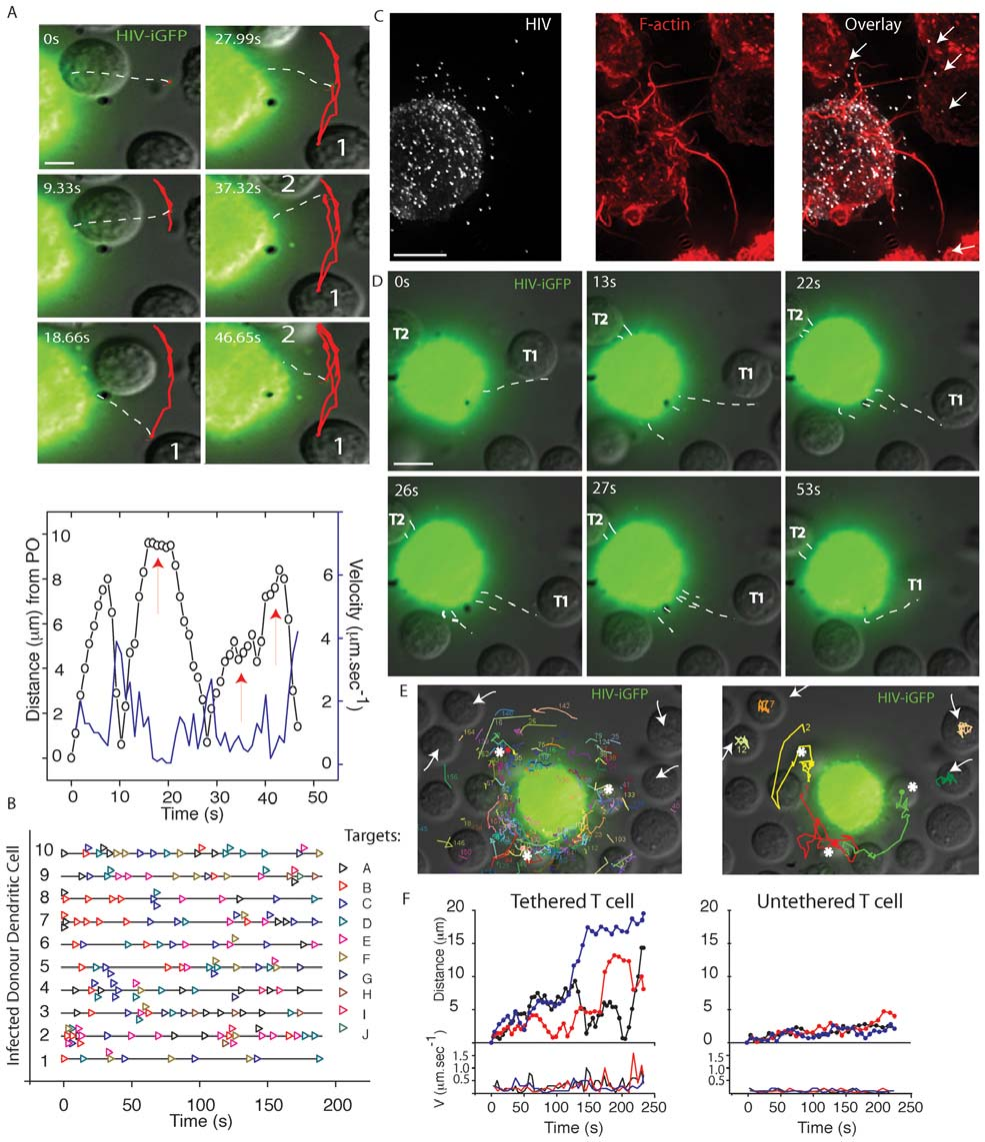
VF contacts and tethering of CD4 T cell targets. To analyze the consequence of VF contact, DCs were infected with HIV-iGFP and subsequently co-cultured with autologous CD4 T cells as outlined in Fig. 1. (**A**) HIV contacts proceed via Arc movements of single VF between cells. In **A.** Upper panel 6 frames have been taken from live imaging time lapse experiment with single particle tracking in red highlighting the movement of VF between two CD4 T cells (labeled 1 and 2). In **A.** lower panel VF velocities (blue) and distance from the point of origin (PO) (black) summarize the movement in the former panel, with CD4 T cell contacts indicated by red arrows. (**B**) Frequency of VF to CD4 Targets over time for 10 representative infected HIV iGFP DC. Each CD4 T cell in the co-culture is listed as “Target” and is represented over time by an open colored triangle (eg. Repeating black triangles, indicated VF contacting the same CD4 T cell “Target A”). CD4 T cell contact data is representative of in excess of 8 independent donors. (**C**)**–**(**F**) Multiple VF co-ordinate to tether CD4 T cells. **C.** Multiple filopodia (highlighted by arrows) are in contact with a neighboring CD4 T cells. Contact is a representative still image, where VF are revealed by F-actin staining using phalloidin (red) and HIV iGFP (white). **D.** CD4 tethering in real-time. Still images have been taken from Video S8 and time is present in seconds in the upper right corners. Viral filopodia have been outlined with white dotted lines as in Fig. 1. Within still images, CD4 T cell denoted “T1” is actively tethered and moved closer to the DC membrane. Whilst CD4 T cell “T2” remains tethered for the duration of the Video. **E.** Multiple VF contacts result in CD4 T cell tethering. In the left panel accumulative VF trajectories for a 3 minute infected DC-CD4 T cell co-culture. White arrows and asterix indicate CD4 T cells where either limited VF contact have occurred over this time (less than or equal to 1) or multiple contacts (>5) have occurred respectively. Image derived from Video S9. In the right panel, trajectories of CD4 T cell over the 3 minute co-culture are presented. Note CD4 T cell repositioning associated with high filopodial activity. Image derived from Video S10. **F.** Distance from the point of origin (upper panel) and velocities (lower panel) for CD4 T cells that are engaged in multiple VF contacts (“tethered”-asterix in E.) versus CD4 T cells with undetectable VF contact (“untethered”-arrows in E.). All data in representative of in excess of 8 independent donors DC-CD4 T cell co-cultures, where greater a minimum of 10 videos were acquired per donor.

VF contacts were either isolated to one to two filopodia engaging in Arc movement between CD4 T cells (Fig. 3A; Video S7), or several VF interacting simultaneously towards CD4 T cell targets (Fig. 3B & C). Overall estimated contacts between infected DCs and CD4 T cells ranged from 260 to 800 per hour; Average contacts = 460±167.hour^−1^; *n* = 45 (D = 3)). Given the speeds of VF movement and the limitations of fluorescence imaging, we must note this imaging represents a contact estimate. Furthermore the restriction of imaging to a limited Z-stack we predict to result in a somewhat conservative contact estimate. Continual and multiple VF activity preceded tethering and subsequent physical movement of CD4 T cells closer to the DC membrane (Fig. 3D–F. Video S8, S9, S10).

### HIV viral filopodia significantly are distinct from HIV cytonemes, nanotubes, and pathogenic actin- tails

**Figure 4:**
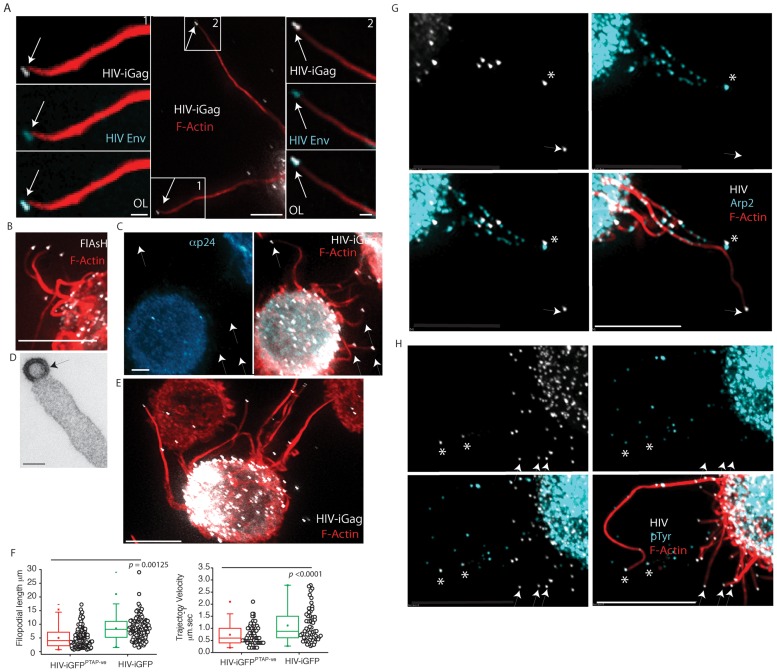
VF are capped and continous with immature HIV buds and do not associate with Arp2/3 antigens. (**A**) HIV envelope staining at the filopodial tip. An infected DC is presented bearing two long VF (HIV iGFP in white and Phalloidin staining in red) in excess of 20 µm (center panel boxed sections labeled 1 & 2). Left and right panels are magnified and HIV envelope stain is presented as blue. Scale bars in the center panel are 5 µm. Scale bars in left and right panels are 1 µm. (**B**)**–**(**D**) HIV at the tip of VF consists of cytosolic uncleave HIV Gag. (**B**) HIV-T FlAsH staining (white) at the tip of VF with F-actin staining using phalloidin (red) (scale bar is at 5 µm). (**C**) Further confirmation that HIV particles consist primarily of uncleaved HIV Gag. Image presented is HIV iGFP DC (all HIV particles will be detected) and counter-stained with the anti-p24/Capsid mAb 183 that specifically detects cleaved HIV p24/Capsid. Note the lack of mAb staining for HIV iGFP particles. Scale bar is at 5 µm. (**C**) Image is representative VF after imaging HIV infected DC via transmission electron microscopy (scale bar at 100 nm). Images in A-C are representative of *n* = 6 infected DC donors with HIV-T, HIV iGFP (fluorescence) or HIV (electron microscopy). (**E**) **&** (**F**) Virions unable to undergo fission from the plasma membrane (HIV GAG PTAP mutants) form VF. (**E**) A representative image of VF is presented in the fluorescent image, with HIV iGFP in white and phalloidin staining of actin in red. Note the mutation of the PTAP motif does not prevent VF formation. Data is representative of three independent infections. (**F**) DCs were infected with HIV iGFP or HIV iGFP^PTAP-ve^. VF lengths and average trajectory velocities are calculated as outlined in Fig. 1 & 2. Statistical significance is presented as *p* values. Data is representative of *n* = 3 independent experiments. (**G**) **&** (**H**) VF are enriched for filopodial antigens, but their antigens do not routinely co-localize with immature HIV particles. Immature DCs were infected as outlined in Fig. 1 and then fixed and counter stained for filopodial antigens (blue), (**G**) Arp2 and (**H**) phosphotyrosine (pTyr) staining are presented as overlays with F-actin (red) and HIV iGFP (white). HIV particles in close association with the antigenic stain are highlighted by asterix in volume projected images, whilst non-associated particles are marked by arrows. Staining for Wasp and Cortactin are presented for comparison in the supplementary Fig. S2. All scale bars are at 5 µm. Images are representative of *n* = 7 independent donors.

For initial phenotyping of viral particles at the tips of filopodia, we first determined whether HIV envelope was present at the filopodial tip. Unfortunately staining of HIV envelope using the 2G12 antibody observed significant signal on the plasma membrane of HIV iGFP infected DCs and U937 cells. Unlike the HIV Gag signal, where there theoretically would be approximately 5000 copies [29], HIV envelope copies have recently been estimated as 14+/−7 trimers per virion [30]. Whilst amplification of the signal using a combination of increased exposure time and CCD/EMCCD camera electronic amplification can permit detection, this was counter balanced by the significant signal of the infected cell membrane. To overcome this, we focused on filopodia that were in excess of 20 µm in length, thereby focusing on a HIV particle that was sufficiently distanced from fluorescence of the cell membrane. Using this approach we could readily detect the presence of HIV Env on the HIV particle capping the filopodial extension (Fig. 4A).

To further phenotype HIV filopodia, we initially determined whether HIV particles were actively forming at filopodial tips. This knowledge would address whether HIV was a part of the filopodia or simply extracellular virus that was bound to the filopodial tip. To determine if HIV Gag had recently originated from the infected cell cytosol (as would be the case for actively budding virions), we utilized HIV-T. Briefly HIV-T relies on the fusion of the FLNCCPGCCMEP peptide to the C-terminus of HIV Matrix within the Gag polyprotein. When the polyprotein is within the cell (during the budding process), the four-cysteine residues are reduced and can bind the cell permeable fluorescent biarsenical dye FlAsH. Once HIV buds from the plasma membrane, the cysteines are no longer reduced and this renders the peptide unable to bind the FlAsH dye [22]. As HIV Gag can be stained at filopodial tips using FlAsH (Fig. 4B), this provided initial support that the particle consists of reduced HIV Gag and thus a HIV particle that was had recently formed. We also confirmed this using HIV iGFP in conjunction with antibody staining using the HIV capsid mAb clone 183. Whilst HIV iGFP detects all HIV (in contrast to HIV-T), we have previously observed the HIV mAb 183 to only detect mature HIV (HIV cleaved Gag) [22]. Using this latter approach, we detect all HIV iGFP positive particles at the tips of filopodia to be HIV mAb negative (Fig. 4C). Both results at the fluorescent level were further confirmed after use of transmission electron microscopy that can resolve immature budding particles and extracellular mature particles by presence or absence of the electron dense capsid core. In the case of VF HIV Gag cleavage had not occurred as the HIV capsid core was not present, thus supporting particles to be budding immature virions that may or may not be in the final stages of fission (Fig. 4D). To further confirm HIV was an immature bud and membrane-associated at the filopodia tip, we generated HIV iGFP with a mutation in the PTAP domain of HIV Gag, as removal of this motif results in lack of viral fission by failure to recruit Tsg101 [31] and thus synchronizes HIV budding particles at the plasma membrane. After DC infection with HIV PTAP mutants, VF length and velocities were lower compared to WT HIV yet comparable VF were observed (Fig. 4E & F; Video S11). Further analysis of total fluorescence of the particles at the tips of filopodia observes a normalized distribution of +/−19.7% (*n* = 110 particles acquired under identical conditions). Therefore given the sum total of all of the abovementioned phenotypic analysis of HIV particles at the tips of filopodia, we conclude HIV particles are incorporated into filopodial tips as immature HIV buds and are not formed by the simple tethering of extracellular mature viral particles.

As particles were virions originating from the cytosol and consisting of immature Gag polyprotein, this immediately differentiated VF from Vaccinia actin tails, as the latter viral particle triggers actin tails from the outside as a cell free viral particle [32]. In addition, as similar filopodia form in uninfected DCs it was important to determine whether HIV was actively involved in filopodial formation or simply embedding at the leading edge of an existing network of filopodia. Thus to further differentiate pathogenic actin tails from VF we sought to detect various antigenic footprints at the tip of filopodia that were characteristic of each pathogen, such as phosphotyrosine epitopes (pTyr) for Vaccinia and Arp2/3 proteins and regulators (Arp2, Wasp and Cortactin) for other Arp2/3 complex dependent pathogenic tails [33]. Therefore, we phenotyped VF on infected DC for pTyr, Arp2/3, Wasp (a leukocyte specific Arp2/3 regulator), and cortactin epitopes. Whilst the majority of each stain was within the cell body, VF were positive for each antigen at focal regions along their length (Fig. 4G & H). However, unlike other pathogenic actin tails, the majority of HIV particles capping VF were not routinely associated with Arp2, Wasp, pTyr, or Cortactin (19%, 16%, 15% and 24%, respectively; *n* = 94 (D = 3); representative images presented in Supplementary Fig. S2). Taken together these data initially support VF to be structures that are divergent from Arp2/3 mediated actin tails, in that there is consistent lack of key cellular antigens in association with HIV particles. However we must note the same cellular antigens were present along the length of VF and thus may indeed be important for the dynamic nature of these structures.

### HIV filopodia formation is dependent on the formin Diaph2

**Figure 5:**
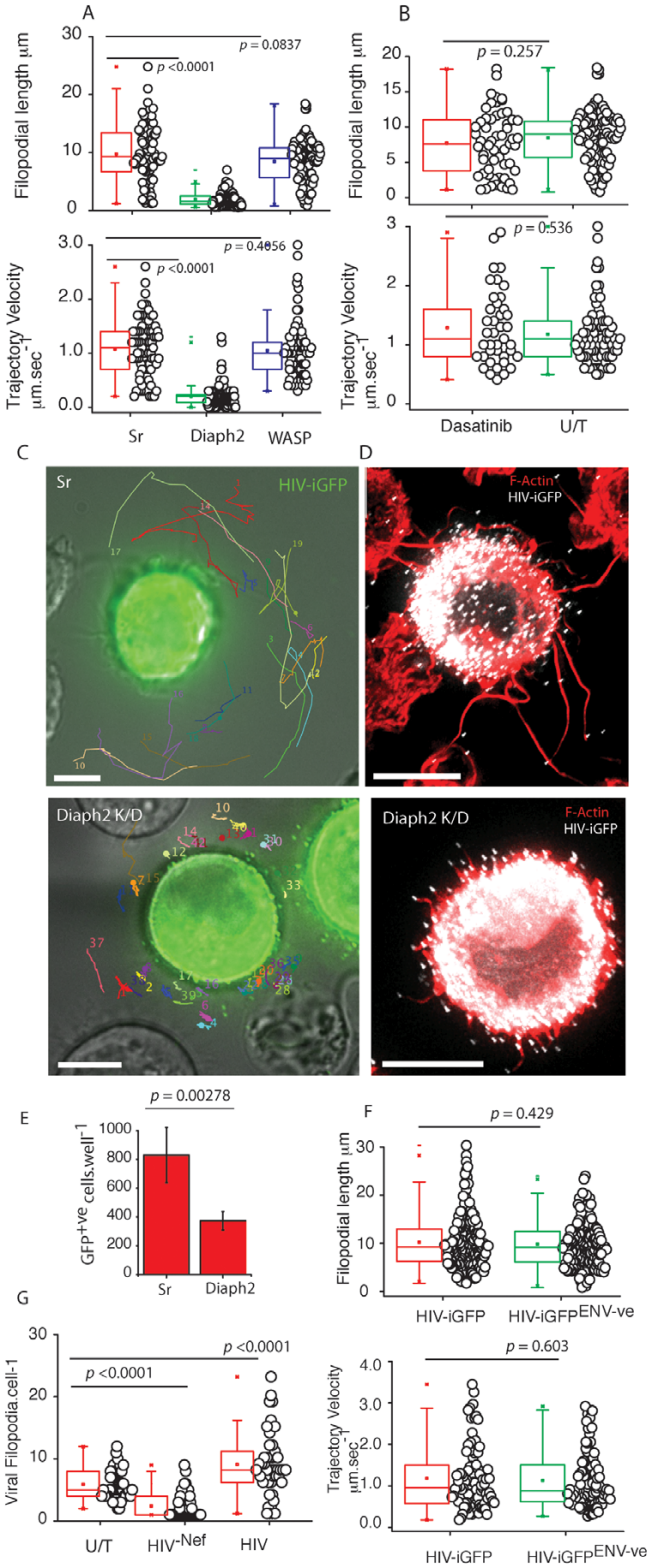
VF form by a Diaph2 dependent pathway with frequency regulated by HIV Nef and not HIV Env. (**A**) To further delineate how VF pathway are formed, Wasp and Diaph2 was knockdown in the U937 cell line using shRNA. After 2 weeks of puromycin selection, resistant U937 cell lines were infected with HIV iGFP and VF lengths and trajectory velocities enumerated as in Fig. 4. *P* values are included to highlight significant differences in each variable. Protein knock-down for Wasp and Diaph2 are presented in Fig. S2E. The house-keeping protein Gapdh is present below to normalize lysate loading. Data is representative of 4 independent infections using HIV iGFP. (**B**) To rule out manipulation of the Arp2/3 filopodial pathway, the U937 cell line was infected with HIV iGFP and two days post infection, infected cells were treated with the Abl/Src kinase inhibitor Dasatinib at 10 µM for 4 hours. Note, under these conditions Vaccinia actin tails do not form (data not shown). VF were then enumerated for lengths and trajectory velocities as outlined Fig. 3. (**C**) Accumulative single particle tracking for 1 minute of VF trajectories in scrambled controls (upper panel) versus Diaph2 knockdowns (lower panel). Note the confined trajectories in the absence of Diaph2. Diaph2 knockdown particle tracking is derived from Video S12. Scale bars are 5 µm. Data from knockdown experiments is representative of 4 independent HIV iGFP infections. (**D**) Fixed cell images of control shRNA (upper panel) and Diaph2 (lower panel) transduced cells infected with HIV iGFP. Note the significantly shorter VF lengths in Diaph2 knockdown U937 cells. Scale bars are 5 µm. Images are representative of 4 independent infections with HIV iGFP. (**E**) Attenuation of cell-cell transfer in Diaph2 knockdown U937. U937 were infected with HIV and 2 days post infection were stained for HIV p24 and enumerated by flow cytometry. After infections were verified to be equivalent, infected U937 cells were co-cultured at a ratio of 1∶5 with the T cell HIV indicator cell line JLTR-R5. Four days post infection, fluorescent images were acquired for the entire well and enumerated using Image J. Standard deviations represent co-cultures in triplicate. Data is representative of 3 independent infections. (**F**) VF form in the absence of HIV envelope. DCs were infected with either VSVg pseudotyped HIV iGFP or HIV iGFP^-ENV-ve^ as outlined in Fig. 1. VF were then enumerated for lengths and trajectory velocities as outlined B. Data is representative of four independent infections. *P* values are presented for significant differences. (**G**) Deletion of HIV Nef leads to significantly lower VF frequency on DC. Enumeration of VF numbers over time in uninfected DCs (U/T), or HIV infected DCS with HIV^−^iGFP or HIV^-NEF^-iGFP. Each point represents live imaging of a VF bearing DC over a period of 2 minutes under imaging conditions outlined in materials and methods. Accumulative data presented is equally drawn from 5 independent donors. Statistical significance is indicated by *p* values.

To explore how VF were formed, we investigated two major pathways of filopodial nucleation/elongation; the F-actin regulators Arp2/Wasp-dependent pathway, and the formin Diaph2 pathway. Whilst initial immunophenotyping observed both Arp2 and Wasp along filopodia, it was difficult to determine in this setting whether they were in fact the dominant regulators of VF. We also attempted immunostaining Diaph2 in a similar manner to Arp2 and Wasp, yet with currently commercially available antibodies, we were unable to specifically detect Diaph2 in fixed samples. Given the limitations of immunophenotyping of filopodia, we generated stable U937 clones expressing shRNA targeting Wasp and Diaph2, and we characterized VF after successful knockdown at the protein level (Fig. S2). When Wasp was knocked-down, VF lengths and velocities remained unchanged compared to the scrambled shRNA controls (Fig. 5A). We obtained similar results when we treated the infected cells with the Bcr/Abl and Src family tyrosine kinase inhibitor Dasatinib under concentrations and conditions that readily prevent Arp2/3 complex activation by Vaccinia (Fig. 5B [34], confirming that Arp2/Wasp was not involved in VF formation/elongation. In contrast, when shRNA knockdown of the formin Diaph2 were used, significantly reduced VF lengths and velocities were observed (Length: 1.95 µm+/−1.683; *n* = 181, versus control *p*<0.0001. Velocity: 0.198 µm.sec^−1^; *n* = 164, versus control *p*<0.0001) (Fig. 5A, C & D; Video S12). Using the same shRNA lentiviral pool, we could not significantly knockdown Diaph2 in primary DCs (Fig. S2F), even after priming DCs with SIV Vpx as previously described [35]. Thus we utilized the TRIPZ lentiviral vector encoding shRNA towards Diaph2, as transduced cells could be identified with the aid of red fluorescent protein. Whilst transduction rates in primary DCs using lentiviral vectors were low, we could identify TRIPZ transduced and HIV infected DC populations. Using this approach, we also observed primary DCs to express significantly shorter VF in comparison to non-transduced and scramble shRNA transduced cultures (2.86 µm+/−1.83 & 9.37+/−3.98 for Diaph2 versus Scrambled shRNA respectively *n* = 79; p<0.0001; Video S13). Although the low primary DC transduction rates using the TRIPZ lentiviral vector limited further study of Diaph2 depleted cells. As the U937 cell line expressed equivalent VF and was homogenously depleted of Diaph2, we utilized this cell line model to dissect the role of VF in cell-cell transfer. Diaph2-dependent VF formation was important for cell-cell transfer as subsequent co-culture of Diaph2 shRNA U937 cells with permissive CD4 T cells line showed significant attenuation in cell-cell transfer compared to control shRNA conditions (control U937 versus diaph2; *p* = 0.00278, *n = 3* Fig. 5E), supporting a direct functional role of long VF in HIV spread. Of note, both stable knockdown of Wasp and Diaph2 in the context of the U937 cell line had no effect on their overall viability, as viability assays using either Alamar Blue or trypan blue exclusion were not significantly different to scrambled shRNA controls. To further control for defects in HIV assembly and release we infected Diaph2 and Sr transduced cells, normalized their infection 3 days post infection to 10% and then harvested viral supernatant 3 days post normalization. Using both detection of reverse transcription (to detect particles in the supernatant) versus infectivity by titering the supernatant on the TZMbl indicator cell line, we did not observe any significant difference in HIV release or infectivity (Supplementary Fig. S2G).

### HIV capping of filopodia is independent of HIV Env, dependent on HIV Gag, and HIV Nef expression increases frequency

To further characterize HIV involved in VF we investigated HIV envelope (Env), Gag and Nef. Initially we focused on Env, given HIV viral cytonemes are exclusively dependent on the presence of Env. To infect DCs with a virus that does not encode Env, we VSVg pseudotyped a HIV iGFP that is genetically devoid of Env. This enabled DC infection and the subsequent appearance of HIV particles in the absence of Env expression. Using this approach, we observed VF lengths and trajectory velocities to be not significantly different with HIV expression Env (Fig. 5F).

As we observed capped HIV at VF tip, we initially hypothesized there was a common link between HIV budding and VF formation. This hypothesis is readily supported by observations that HIV Gag membrane targeting (that proceeds budding) and filopodial formation occur at the same phosphoinositol 4,5-bisphosphate (PIP_2_) plasma membrane domain [36], [37]. Alternatively filopodial formation requires outward membrane curvature generated by F-Bar proteins (eg. Toca-1) [38] and the formation of the HIV bud may provide the equivalent “membrane curve” substitute leading to the seeding of filopodia at the membrane during HIV budding. To test this potential “common lipid domain” hypothesis, we reasoned that HIV Gag expression alone should result in VF capping. We generated a plasmid that would allow expression of the same HIV Gag -iGFP polyprotein that is expressed in the HIV iGFP virus, and we generated stable HeLa cell clones that express Lifeact-mcherry [39], can be used to readily visualize F-actin and VF dynamics in real-time. The choice of HeLa cell was motivated by its ease of transfection within our Gag -iGFP construct and the fact filopodia can form using this adherent cell type. Through live imaging of F-actin and HIV Gag expression alone, we readily observed the formation of VF (Video S14). Thus localization to the filopodial tip, is a function of HIV Gag alone, and is entirely consistent with Gag embedding at the same lipid site, where VF are nucleated. Finally we investigated the role of the HIV accessory gene Nef as it has recently been observed to influence non-viral filopodial formation on CD4 T cells [40]. We generated deleted HIV Nef in HIV iGFP. Using live cell imaging, we observed significantly greater numbers of VF in HIV^WT^-iGFP versus their Nef deleted counterparts (termed HIV^NEF-ve^-iGFP) (Fig. 5G; HIV^WT^-iGFP = 8.89±5.17 VF.cell^−1^ versus HIV^NEF-ve^iGFP = 2.49±2.01 VF.cell^−1^, *p*<0.0001). Although uninfected DCs expressed similar filopodia to infected DCs, their frequency compared to VF in HIV infected cells, was significantly lower (Fig. 5G). Therefore whilst HIV Gag localizes the virus to the tip, expression of HIV Nef positively influences VF frequency. To further determine whether HIV Nef alone can increase filopodial numbers, we inserted the HIV *nef* gene into the lentiviral vector pRRLSIN.cPPT to create Nef-eGFP fusion equivalent to that used by Nobile and colleagues [40]. Using this approach we could readily transduce primary DCs with the Nef-eGFP fusion protein and its matched eGFP control. Expression of Nef-eGFP alone did not significantly increase the frequency of filopodial expression compared to eGFP only transduced DC co-cultured with autologous CD4 T cells (2.43+/−1.31 versus 2.875+/−1.67 filopodia.cell^−1^ for Nef-eGFP versus eGFP respectively; *n* = 49, *p* = 0.4165).

### Viral filopodia, converge to form viral synapses

**Figure 6:**
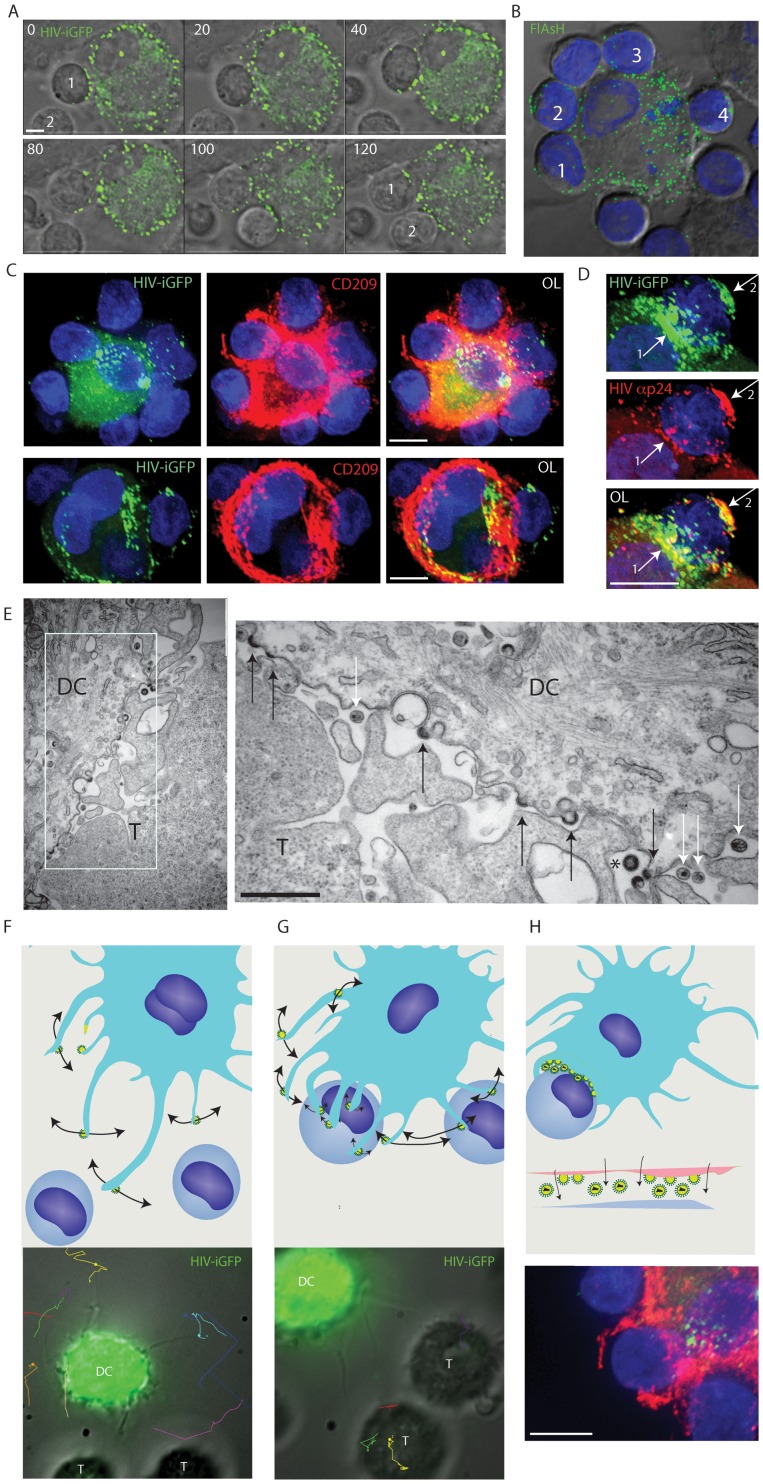
Viral filopodial contacts precedes the formation of an enveloping DC-CD4 T cell contact. (**A**)**–**(**D**) Long-term contact by an infected DC leads to enveloping of the neighboring CD4 T cells. (**A**) Long-term enveloping in real-time. To follow the consequence of initial VF contact and tethering, HIV iGFP infected DC initially engaged in VF contacts with CD4 T cells were imaged over a period of 2 hours. Stills were taken from Video S15, with time elapsed presented in the upper left corner as minutes. In the final frame, two CD4 T cells are labeled 1 & 2 that have been enveloped by the infected DC. Scale bar at 5 µm. (**B**) Enveloping of multiple CD4 T cells. DCs were infected with HIV-T and 5 days post infection co-cultured with CD4 T cells at a ratio of 1 DC to 3 CD4 T cells for 2 hours. HIV particles were then imaged using FlAsH staining (green), samples fixed/permeabilized and nuclei countered stained with DAPI (Blue). Note CD4 T cells numbered 1 through to 4 have distal and proximal FlasH positive viral particles across their membrane. (**C**) Viral synapses between infected DC and CD4 T cells mature into extensive enveloping contacts. DC-T cell co-cultures are generated as per conditions in B, with the exception of the use of HIV iGFP (green). After 2 hours of co-culture, cells were stained with the DC membrane marker CD209 (red) and nuclei counterstained with DAPI (blue). Upper panel is representative of an infected DC engaging multiple CD4 T-cells as in B. In the lower panel, the infected DC is limited to two CD4 T cells, with the T cell conjugated on the right with a representative infected DC-T cell viral synapse. Scale bars are at 5 µm. Images are representative from 4 independent donors. (**D**) Long-term enveloping precedes viral fission across the synaptic cleft and seeding of the CD4 T cell with mature HIV. DCs were infected and co-cultured with autologous CD4 T cells as in (C). Total HIV (iGFP - green), mature HIV (Anti-p24/Capsid clone 183 in red) staining was carried out as per materials and methods. Nuclei are stained with DAPI as in (B). Arrow denotes accumulation of mature HIV particles at the viral synapse and also at the distal CD4 T cell membrane. Note HIV iGFP staining at the viral synapse exceeds mature HIV particles. Scale bar at 5 µm. (**E**) Electron microscopy confirms the presence of both immature/actively budding (black arrows) and mature HIV virions (white arrows) between the DC-T cell mature contact zone. The appearance of a rare immature HIV particle is marked by *. Scale bar, 500 nm. Data from A–D is representative of *n* = 5 donors. (**F–H**) Schematic of the sequential contacts by VF (**F**), leading to selection and tethering (**G**) and enveloping of the T cells target coinciding with viral release (**H**). Whilst upper panels are schematics, lower panels have included primary representative data to support schematics.

The capacity of VF to mediate tethering and co-ordination of neighboring CD4 T cell contacts supports their role as viral synapse (VS) precursors, with VS formation triggered by HIV envelope and eventually mediating CD4 T cell infection. To image closer synapse formation, we identified infected DC with VF in close proximity to CD4 T cells and imaged the potential for synapse formation. Using both and HIV-iGFP (Fig. 6A) and HIV-T (Fig. 6B), we observed viral transfer across to the CD4 T cell membrane. We observed dynamic movement of newly produced HIV virions (HIV-T and HIV iGFP positive) over the entire CD4 T cell membrane (Fig. 6A & 6B; Video S15). Viral movement was over and around the CD4 T cell membrane (Video S15), with continual seeding and repositioning of the virus for up to 3 hours (the duration of the video acquisition). In addition, this phenomena could proceed from one infected DC over multiple CD4 T cells simultaneously (Fig. 6B & C). Of note co-culture of CD4 T cells with DCs infected with VSVg HIV^ENV-ve^ (HIV envelope negative) did not result in seeding of virus over the CD4 T cell surface. Whilst HIV iGFP would detect all virions, the detection of HIV-T on the CD4 T cell membrane supports either covering of the CD4 T cell membrane by DC in a form of cytophagocytosis or assembly, release and dissemination of HIV from the DC cytosol (as HIV-T only stains reduced cytosolic HIV Gag). To determine the extent of DC membrane that cover CD4 T cells at the viral synapse, we fixed and stained HIV infected DC-CD4 T cell co-cultures with the abundant DC membrane antigen CD209. Whilst the DC membrane covered substantial surface areas of the CD4 T cells engaged in viral synapses, the distal areas of the CD4 T cell membrane were not CD209 positive and thus we conclude although CD4 T cells were engulfed, they were not cytophagocytosed (Fig. 6C).

Although our results confirmed that VF eventually mature into a close stabilized contact that extensively covered the CD4 T cell membrane, the actual point of viral fission, maturation and CD4 T cell infection was still not known. Thus utilizing antibodies specific for mature HIV [22], we observed distal, yet frequent, VF contacts with CD4 T cells only bearing immature HIV buds. However, when analyzing closer DC- CD4 T cell contacts we observed the appearance of mature HIV particles not only at the proximal contact of the synaptic cleft, but also at the distal sections of the CD4 T cell plasma membrane (Fig. 6D). We further analyzed the proportion of mature HIV particles by scoring mature HIV particles by antibody staining using the anti-capsid mAb 183 versus total HIV using HIV iGFP. Using this approach we observed the proportion of mature HIV virions at the synapse to be 0.4282+/−0.272 (for *n* = 56 synapses). In contrast, at the distal CD4 T cell membrane, the mature particle proportion was 1.6+/−1.462 (for *n* = 56 synapses). Taking the accumulative observations from live cell imaging of DC-T cell viral synapses and analysis of mature HIV particles, we initially conclude at the synaptic cleft the majority of particles appearing consist of immature HIV buds that eventually undergo fission, viral maturation and trafficking to the distal side of the CD4 T cell membrane. Further ultrastructural analysis of virus at the DC-T cell synaptic junction confirmed this observation with the majority of HIV particles appearing at the synapse consisting of HIV buds (58%), followed by mature HIV particles (35%) and on rare occasions immature particles that have undergone fission (7%) (Fig. 6E) (*n* = 120 particles counted at DC-T cell synaptic junctions).

### Abundant expression of HIV viral filopodia correlates with the efficiency of DC to transfer HIV to CD4 T cells from the de novo pool

**Figure 7:**
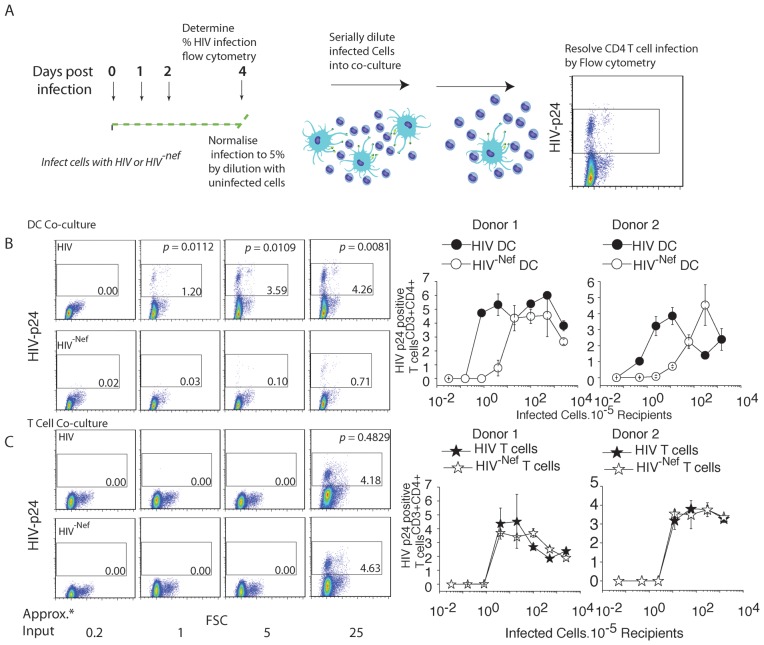
High VF frequencies correlate with the efficiency of immature DC viral transfer. (**A**) Schematic of the DC-CD4 T cell transfer assay. DC are infected with either HIV^WT^ (high VF frequency) or HIV^-NEF-ve^ (low VF frequency) pseudotyped with the VSVg glycoprotein, to ensure equal infection frequencies. After 4 days, DC infections are normalized to 5% with uninfected DC. Normalized populations are serially diluted below 1 infected DC per co-culture. 4 days post co-culture, CD4 T cell infections are resolved by staining cells for of HIV capsid and resolution by flow cytometry. (**B**) Flow cytometry detection of HIV p24 within CD4 T cell recipients when input infected DC are limiting (upper panel) versus (**C**) when input infected CD4 T cells are limiting (lower panel). Approximate infected cell number input into co-cultures is indicated at “Approx. Input*” on the X-axis. CD4 T cell infection frequencies are detected by the accumulation of a high HIV p24 population as indicated by the square gate in each dot-plot. Statistical difference is presented in upper HIV WT panels, and is calculated from data acquired from the same assay performed in triplicate. CD4 T cell infection frequency versus infected donor input is further summarized in right panels for two representative donors. Standard deviations represent co-cultures in triplicate. Data is representative of *n* = 8 independent donors.

The significantly lower VF number in HIV^NEF-ve^-iGFP infected DCs gave the unique opportunity to determine the potential role of VF in absolute viral transfer efficiency in primary DC, whilst not significantly influencing their viability and/or phenotype. Thus we tested the ability of immature DC infected with HIV^NEF-ve^(low VF frequency) versus HIV^WT^ (high VF frequency) to transfer virus to a permissive (activated) autologous CD4 T cell population. Given CD4 T cells are VF low/absent expressing cell types, we also tested CD4 T cell to CD4 T cell transfer as a control. We stringently normalized both HIV^NEF-ve^ and HIV^WT^ infected populations (Fig. 7A schematic) prior to dilution and addition to target CD4 T cell populations. After 5 days co-culture, we utilized flow cytometry to not only resolved infected T cell population, but also determine the proportion infected. To enumerate the latter we take advantage of HIV capsid staining using the directly conjugated mAb clone KC57. In infected populations (both DCs and CD4 T cells), HIV capsid accumulates to significantly high levels to enable resolution of uninfected and infected populations as previously described [16]. Resolution of productive infection (versus only carriage of the virus) is further supported by the down-regulation of CD4 only observed in the capsid high population [11]. To unequivocally rule out this population is a result of CD4 T cell Gag acquisition independent of infection, we incubated DC-T cell co-cultures with 10 µM of AZT and confirmed the appearance of the p24/Capsid high population to be a result of productive infection (Fig. S2H).

At limiting dilutions (the equivalent of adding 1 to 25 infected cells per 100,000 CD4 T cells; Fig. 5D), HIV^WT^ -infected DC were significantly more efficient at virus dissemination than both DCs infected with HIV^NEF-ve^ and CD4 T cells infected with HIV^WT^ (Fig. 7B). In contrast there was no significant difference in efficiency of dissemination when HIV^WT^ or HIV^NEF-ve^ HIV infected CD4 T cells were the viral donors (Fig. 7C). These data coupled with lower HIV transfer in Diaph2 knockdown U937 transfer, suggest that efficiency of viral spread is correlated with high frequency of long dynamic VF.

## Discussion

The key to HIV survival is its ability to find new targets and to do so within an increasingly hostile environment, due to the progressive activation of the acquired immune response. Limiting DC numbers can seed CD4 T cell populations in a manner analogous to how they stimulate the immune response, thus infected DC represent a point of viral amplification within a CD4 T cell population. Herein we have mapped key mechanisms of how HIV spreads from this important cell type. In DC, HIV has hard-wired itself into the tip of promiscuous contacting filopodia. Initially VF abundance, nature of movement, and velocity allow HIV to partake in hundreds of fleeting CD4 T cell contacts. Once CD4 T cells are tethered by VF, they are then subsequently repositioned and converge to become the DC-T cell viral synapse. The mechanistic transfer of virus via long viral filopodia is generated by the formin Diaph2, and not via the Arp2/3 complex pathway commonly observed with other pathogens in the generation of actin tails. In addition HIV Nef positively influences VF frequency and also coincides with the ability for DC to spread virus to CD4 T cells. Thus herein we hypothesize the potency of viral dissemination by DC to be the combination of CD4 T cell target selection through a mechanism of repertoire scanning by long Diaph2 dependent filopodia, followed by the maturation of this contact to ensure the virus not only has access to the maximum surface area of the CD4 T cell membrane, but is also released when the virus is least exposed to the extracellular environment.

VF are not variants of nanotubes or viral cytonemes and there is significant evidence herein that set them as unique structures in their own right. Their ability to form in the absence of HIV envelope, their formation on infected cells, lack of continual cell-cell tethering and terminal restriction of the virion to the VF tip and no evidence of movement along the filopodia are in immediate opposition to the formation and function of viral cytonemes/nanotubes. Furthermore, VF formation is dependent on the formin Diaph2, a key regulator of long filopodia [41] and an actin regulator enriched in cells of myeloid lineage [42]. We must also clarify that the structures described herein on immature DC are not equivalent to that recently described by Nikolic and colleagues [43]. Briefly, Nikolic et al, observed outside in signaling by HIV envelope by engagement of the C-Type lectin CD209, leading to activation of the Src-CDC42-Wasp pathway. This culminated in membrane extensions originating from DC being bound with mature HIV extending towards target CD4 T cells. VF on infected DC differ significantly from such membrane extensions. Firstly, HIV DC membrane extensions represent early transfer of HIV *in trans* and not via the *de novo* reservoir of infected DCs as observed for VF. Secondly, mature virions appear along the length of membrane extensions as opposed to newly forming virions exclusively located at the tips of VF. Finally, membrane extensions are dependent on HIV envelope signaling through a Src-CDC42-Wasp pathway, whereas VF appear both independent of HIV envelope, in cells depleted of Wasp and in the presence of saturating levels of the Src inhibitor Dasatinib.

As aforementioned, the Arp2/3 complex has been implicated in the formation of pathogenic actin tails for Vaccinia, enteropathogenic Escherichia coli, Listeria, and Shigella [33], [44], [45], [46]. We could not conclude that the Arp2/3 complex was the dominant cellular regulator involved in HIV VF. This was supported by the lack of Arp2 and phosphotyrosine association with HIV at filopodial tips, and the inability of the Abl/Src kinase inhibitor Dasatinib and Wasp knockdowns to disrupt VF length and trajectories. Although Diaph2 is essential for elongation of long filopodia in our study and elsewhere [41], the persistence of often numerous short VF (similar in nature to that recently described filopodia in cryo-electron microscopy studies of HIV Gag expressing U87mg cell line [47]) does not support initial nucleation of VF at the plasma membrane to be Diaph2 dependent. Thus, the initial nucleation of shorter VF, that we would predict to be the foundation of longer VF driven by Diaph2, remain currently unknown. As HIV Gag alone locates to the tip of VF, there may be key variables intrinsic for HIV budding that are also important in VF formation. The use of a common plasma membrane lipid by HIV and filopodia may be one explanation of VF capping [36], [37] and the appearance of similar filopodia in uninfected DCs when co-culture with autologous CD4 T cells is consistent with this hypothesis (HIV budding at filopodial birth sites). Although the high frequency of HIV positive filopodia in infected DC would mean HIV buds near completion would need to occupy greater than 90% of lipid sites where filopodia arise. The alternative explanation would be HIV can influence filopodial formation by either physically changing the membrane or via the recruitment of a protein involved in actin regulation, analogous but obviously divergent to other pathogens. For the former hypothesis, the formation of the HIV bud may substitute for F-Bar containing proteins (eg. Toca-1) to generate the membrane curvature needed for seeding F-actin polymerization that leads to filopodia. For the latter hypothesis, the common theme of all pathogens that cap filopodial-like structures is the use of a pathogen encoded protein to regulate actin (eg. A36R, ActA and Sca2; Reviewed in [20]) and thus this would support a viral protein positively influencing the formation of filopodia. HIV Gag has been previously observed to recruit cellular proteins involved in the endosomal sorting pathway, such as Tsg101. However HIV Gag PTAP mutants did not support VF to be dependent on this cellular pathway. Whilst HIV Gag alone may influence VF formation, we also observed a positive influence of HIV Nef in their formation. Initial studies of SIV transfer from exposed DC to CD4 T cells [48] and latter studies using HIV [49] showed HIV transfer phenotypes from immature DC that are consistent with our current observations. Whilst other mechanisms have been suggested for the ability of Nef to manipulate immature DC [50], [51], at present we favor the hypothesis of Nef expression leading to greater VF numbers and in turn greater viral spread from DC. Further support for this hypothesis is observed in Diaph2 knockdown experiments, in which the appearance of shorter static filopodia also results in significant attenuation of cell-cell viral transfer. However Nef expression alone does not mediate the high frequency VF phenotype. Rather this phenotype only appears when Nef is expressed in the context of the viral genome. Thus we presently support the mechanism of increase in filopodial numbers to be a synergy between HIV Gag and Nef within the cytosol of infected cells that positively influences VF frequency.

Prior to contacting CD4 T cells, we observed VF to engage in non-random fast and broad overlapping sweep targeted trajectories. Multiple VF in concert effectively increase the contact zone and thus the probability of contacting a neighboring cell type. For instance we currently estimate the number of T cells that fit within a contact zone maintained by several filopodia at 10 µm to be 104 T cells per infected DC (accounting for the void volume between spheres as outlined by Hale [52]). In contrast, the DC plasma membrane can only maintain upwards of 14 T cells bound directly. Thus increasing the contact zone for the virus is one advantage, but moving the viral particle away from the plasma membrane may be additionally advantageous. For instance, this would prevent localized viral budding that would otherwise lead to re-infection (superinfection) of the viral producing cell (DC) or, in the context of an antigen presenting cell, antigenic processing and viral degradation. Whilst recent studies have observed cell-cell contact and mass viral release to result in evasion of HIV antiretrovirals [3], the dynamics of DC to T cell transfer may provide further obstacles in blocking cell-cell transfer. For instance, the initial contacts and tethering of CD4 T cells by VF can proceed in the absence of HIV envelope expression and potentially may negatively influence the potency of HIV attachment/fusion inhibitors and/or neutralizing antibodies.

The eventual synaptic contact between infected DC and CD4 T cells is intriguing as a significant proportion of the DC membrane is involved in the formation of the viral synapse. As a consequence, rather than a synaptic-like button forming, as has been observed for CD4 T cells [53], the viral synapse resembles a “cup’ where a large surface area of the CD4 T cell membrane is engaged. Shortly following DC-T cell engagement dynamic seeding of virus proceeded over the membrane of the CD4 T cell, where a significant proportion of HIV buds appeared at the DC-T cell contact point followed by the appearance of mature virus accumulation at the distal face of the CD4 T cell membrane. The extensive sheet-like contacts and the envelope dependent dynamic viral seeding of CD4 T cells are reminiscent of recent work by Yu *et al* and Felts *et al*, [10], [54], who characterized similar sheet-like contacts occurring between mature HIV pulsed DCs transferring virus in the process of *in trans* CD4 T-cell infection. However the important difference to note in the latter studies was the fact the mature DCs were not infected, but rather transferring a finite pool of mature virions to a contacting CD4 T cell population. Infected DCs on the other hand engage CD4 T cells in a similar manner but continually supply new virions through HIV budding at the at the DC-T cell synapse. However it is important to note that both mature DCs and infected immature DCs are efficient at seeding CD4 T cell populations via the above mentioned mechanistically distinct transfer pathways.

To conclude, in observing the real-time dynamics of *de novo* HIV spread between infected DC and CD4 T cells, we have identified a novel pathogenic structure, that combines the biogenesis of newly budding HIV virions with that of long Diaph2 dependent filopodia. Whilst projection of virus away from infected DC has the immediate benefit of preventing superinfection and viral degradation, VF dynamics support a structure that has corrupted a pre-existing and ubiquitous cell–cell contact pathway needed for the communication of the primary immune response. Fast and numerous contacts by VF ensures neighboring cells are filtered, leading to target selection and sychronized delivery of virus *en masse* and at a time where cell-cell membrane contacts are not only maximal, but provide limited access to the hostile external environment.

## Materials and Methods

### Constructs for live imaging

Plasmid constructs were all based on the CCR5 using HIV pNL43^AD8ENV^ clone (Courtesy of E.Freed, through the AIDSreagent Repository). As all viruses are derivatives of NL43^AD8ENV^, they are referred to hereon as “HIV”. HIV^ENV-ve^ was generated from the pNL43 clone as previously described [55]. HIV^NEF-ve^ clones were generated by sub-cloning the BamHI-NcoI fragment of HIV NL43 into pLitmus29 (New England Biolabs, Beverly, MA) with mutagenesis of the Nef start codon to a stop codon carried out using the Quickchange mutagenesis method (Strategene, La Jolla, CA). To insert imaging labels into the HIV genome (for either HIV-T or HIV iGFP, the SpeI-BssHII restriction fragment from pNL43 was sub-cloned into pLitmus29 and all subsequent mutations were carried out using the Quickchange mutagenesis. All details regarding HIV-T and related biarsenical staining have been previously described in detail [22], [56]. The introduction of the eGFP sequence into HIV Gag open reading frame has been previously described [23] and all viral variants listed as HIV iGFP are equivalent in Gag sequence to that previously described by Hubner and colleagues as HIV iGFP. After insertions into the GAG polyprotein were made in the pLitmus vector, clones were subsequently verified by sequencing and re-introduced into pNL43^AD8ENV^, pNL43^ENV-ve^, pNL43^AD8ENV, NEF-ve^ and pNL43^AD8ENV, PTAP-ve^ (HIV PTAP mutant courtesy of Dr Eric Freed, NIH, USA) using the SpeI-BssHII specific restriction sites. All transfections to generate HIV stocks used polyethylenimine (at 1 mg.ml^−1^ and neutralized to pH 7 (PEI Max, Polysciences, PA)) transfection of the F293T cell line (Invitrogen, Carlsbad, CA) as previously described [22]. Briefly, 20 mg of total plasmid DNA is diluted into a final volume of 1 ml in tissue culture grade 0.9%w/v NaCl (Sigma). 80 ml of PEI Max, as prepared above, is then added drop-wise to the diluted plasmid DNA & vortexed for 10 seconds. The subsequent Plasmid-DNA mix is then left to stand at room temperature for 10 minutes and then added drop-wise to 30×10^6^ trypsinised F293T cells resuspended in a final volume of 15 mls DMEM supplemented with 10% Fetal calf serum. Cells were cultured overnight in a T-150 tissue culture flask after which cells were gently washed and new DMEM media was replaced. For rescue experiments of HIV iGFP with either HIV or psPAX2, molar ratios of HIV iGFP to the respective plasmid were generated whilst maintaining the total DNA content. To ensure PEI∶DNA ratios did not change (for psPAX2 rescue experiments), all reactions were normalized to 20 mg using molecular grade salmon sperm DNA (Sigma). For instance a ratio of 1∶1 HIV iGFP to HIV was typically 10 mg of HIV iGFP to 10 mg of HIV, whereas a 1∶2 ratio was 6.7 mg of HIV iGFP to 13.3 mg of HIV. Unless otherwise indicated, to generate “rescued” HIV iGFP viral stocks, HIV iGFP was co-transfected with psPAX2 (From Didier Trono through the AIDSreagent, NIH. NIAID) at a molar ratio of 2∶1 HIV iGFP to psPAX2. DC infections with HIV^ENV-ve^, utilized VSVg-envelope pseudotyping as previously described [22]. Routine production, purification and titering of viral stocks are outlined as previously described [22].

To image F-actin dynamics the optimized LifeAct sequence [39] was fused to the N-terminus of mCherry with the pLVX-mCherry lentiviral vector (Clontech, Mountain View, CA). Lentivirus was the generated as previously described [57] using polyethylenimine (Polysciences) and the lentiviral helper plasmids pHEF-VSVg (Courtesy of Dr. Lung-Ji Chang through the AIDsreagent repository, NIH) and psPAX2. The HIV permissive HeLa cell TZMbl (Courtesy of Dr. John C. Kappes, Dr. Xiaoyun Wu and Tranzyme Inc, was then infected at an MOI of 1 and 7 days post transduction, high Lifeact mCherry single cells clones were sorted into 96 well plates using a FACSAria (Becton Dickinson, San Jose, CA). TZMbl stably expressing LifeAct-mCherry we now refer to as TLC cells.

To image HIV Gag alone with iGFP equivalent to the HIV iGFP construct, we generated the TI3 plasmid by placing the HIV GAG-iGFP open reading frame over the existing eGFP open reading frame in the pEGFP-NI vector using the Kpn1 and Not1 restriction sites. To image the formation of virus like particles using the TI3 plasmid, we co-transfected 1×10^6^ TLC cells with TI3 and psPAX2 at a ratio of 0.5 mg∶0.5 mg with 9 ul of polyethylenimine (Polysciences PEI Max prepared as described prior). One day post transfection, cells were trypsinized and replated in of a 35-mm imaging dishes with #1.5 coverslips (MatTek, Ashland, MA), and cultured for a further 24 hours prior to imaging.

To express HIV Nef in isolation, we PCR amplified the HIV *nef* gene from the pNL43 plasmid (AIDSreagent) using oligonucleotides AATTCTAGATGGGTGGCAAGTGGTCAA & CGGAGTACTTCAAGAACTGCTGGGATCCTAT. Amplicons were then purified and restriction digested with XbaI and BamHI and directionally ligated into the XbaI/BamHI cut pRRLSIN.cPPT lentiviral vector (courtesy of Didier Trono via the Addgene plasmid repository). Note the above lentiviral vector was modified to encode the XbaI restriction site 6 base pairs upstream from the BamHI site. Vectors were then sequenced to ensure no PCR driven base pairs were changed in the cloning process. Lentiviral particles were then generated using the helper plasmids pHEF-VSVg and psPAX2 as described for LifeAct-mCherry lentiviral particles. To transduce DCs were generated SIV 3+ virus like particles (SIV 3+ is courtesy of Andrea Cimarelli) as previously described [35] and exposed DCs 24 hours prior to lentiviral transduction. Control experiments transduced primary DCs with eGFP only pRRLSIN.cPPT lentiviral particles. DCs were imaged after 4 days post-transduction under live imaging conditions outlined for HIV iGFP infected DCs.

### Cell culture and infection

All transfections to generate viral stocks use the HEK F239T cell line (Invitrogen) are described in detail above and else where [22]. Initial titering and analysis of HIV viral stocks utilized the TZM-bl indicator cell line. Briefly, this cell line expresses HIV LTR-b-galactosidase and infection is detected 4 days post infection using X-gal staining and enumeration of infected cells using an Elispot reader (AID Diagnostika) as previously described [58]. Purified monocytes were isolated from peripheral blood mononuclear cells (PBMC) using CD14 positive selection as outlined by the manufacturer (Miltenyi Biotech, Gladbach, Germany) with the exception of using 4°C PBS supplemented with 1% (v/v) human serum (Sigma) and 1 mM EDTA (Sigma, St Louis, MO). Post isolation, monocytes were cultured with IL-4/GM-CSF (Biosource, Invitrogen), 400 U/1000 U.ml^−1^) supplemented RPMI-1640 media (Invitrogen) containing 10% fetal calf serum (Invitrogen) media. Unless otherwise stated, all DC infections were at 1.00×10^5^ TCID_50_.10^−6^ cells as previously described [22]. Four days post infection % frequency of DC infection was determined by flow cytometry detection of HIV Gag (p24/capsid) staining of cells with clone KC57 (Beckman Coulter, Miami, FL) and gating on the resolved p24^hi^ population. To confirm the appearance of *de novo* HIV Gag in the p24^hi^ population, the nucleoside reverse transcriptase inhibitor AZT was also included as a control. To ensure *in vitro* derived DC were not exposed to any maturation stimuli (eg. Endotoxin contamination) during culture, surface phenotyping of CD206, CD209, CD83, CD25 was routinely carried out as previously described [59]. Autologous CD4 T cells were isolated from the CD14 depleted fraction of PBMC by depletion using the CD4 T cell Isolation Kit II as outlined by the manufacturer (Miltenyi Biotech). CD4 T cells were subsequently cultured at a density of 2×10^6^ cells.ml^−1^ in RPMI 1640 supplemented with 10% fetal calf serum and 20 U.ml^−1^ IL-2 for four days, after which they were counted and co-cultured with infected DC populations at a ratio of 3 CD4 T cells to 1 DC for live imaging. For quantitative co-culture assays, purified CD4 T cells were activated with T Cell Activation/Expansion Kit as described by the manufacturer (Miltenyi Biotech). Purity and activation status of CD4 T cells were determined by CD4, CD3, CD69 and CD25 surface staining and flow cytometric analysis.

### Live and fixed imaging of HIV iGFP DC infected populations

Live-cell imaging was carried out using the 60× 1.42 NA oil immersion lens with an inverted Olympus IX-70 microscope (DeltaVision ELITE Image Restoration Microscope, Applied Precision/Olympus) and unless otherwise stated a photometrics CoolSnap QE camera. For DC-CD4 T cell co-culture movies, 1×10^5^.200 ml^−1^ cells were cultured in the centre of a 35-mm imaging dishes with #1.5 coverslips (MatTek) prior to adding 2 mls of warm GM-CSF/IL-4 supplemented media (as above). Imaging was carried out 30 minutes after co-culture, once cells has settled above the coverslip within the Matek dish. For time-lapse movies, eGFP and DIC channels were imaged at approximate 3 frames.sec^−1^, with time-lapse movies presented as overlays. Manual single-particle tracking was performed using ImageJ (NIH, Bethesda, MA) using the MtrackJ plugin (courtesy of Erik Meijering, at Erasmus MC - University Medical Center Rotterdam). VF tip Velocities were calculated for each particle based on movement across the XY focal plane (ie. If VF tips moved out of the focal plan by movement in Z direction, they were excluded for velocity calculations). Arc velocities were measured when VF were not in contact with CD4 T cells, whilst scan speeds were calculated when VF tips were in association with neighboring CD4 T cells. Hurst Exponents were calculated from trajectories that were in excess of 20 seconds of duration (that is, the VF that remained within the focal plane during the trajectory period). To calculate approximate contacts over time, fields of view with equivalent cellular densities were selected for analysis and VF contacts with neighboring CD4 T cells were enumerated over a 10 minutes period, with results presented as approximate contacts per hour.

For fixed cell imaging, cells were resuspended at 2×10^6^ cells.ml^−1^ in warm RPMI 1640, cytospun onto 22×60 mm 1.5 coverslips (VWR international, Batavia, IL) pre-coated with Celltak (BD Bioscience), and then fixed in 4% paraformaldehyde (Sigma) for 20 minutes at room temperature and then neutralized with 50 mM NH_4_Cl (Sigma) for 3 minutes. Cells were permeabilized with 0.05% Triton-X (Sigma) for 1 minute at room temperature, stained with the indicated mAbs in presence of 5% appropriate species serum, followed by the appropriate secondary reagent. Murine mAb were used at 5 mg.ml^−1^ unless otherwise stated. Arp2 mAb (clone FMS96) was from Abcam (Cambridge, United Kingdom), Cortactin mAb (clone 4F11) and anti-phosphotyrosine (Clone 4G10) were from Millipore (Billerica, MA) and WASP mAb (Clone D1) was from Santa Cruz Biotech (Santa Cruz, CA). HIV p24 mAb was clone 183 (Courtesy of Dr. Bruce Chesebro and Kathy Wehrly via the NIH AIDSreagent program). CD209 mAb DCN46 was from Becton Dickinson (Franklin Lakes, NJ). The human monoclonal antibody to HIV-1 gp120, 2G12 was courtesy of Dr. Hermann Katinger through the NIH AIDSReagent program. Secondary antibodies were goat anti mouse or goat anti human Alexa Fluor 555 (IgG H+L; Highly cross-absorbed; Invitrogen). All washes were performed in PBS supplemented with 1% fish skin gelatin (Sigma) and 0.02% saponin (Sigma). After staining, coverslips were counterstained and mounted with Prolong gold anti-fade reagent with DAPI (Invitrogen) onto glass slides. Cells were visualized through a 100× 1.4 NA oil immersion lens with an inverted Olympus IX-70 microscope (DeltaVision Core) and a Photometrics CoolSnap QE camera. Images were acquired as 50 to 60 serial optical sections of 0.15 µm to 0.2 µm then deconvoluted and volume projections of the entire Z-series were generated using DeltaVision SoftWoRx software, version 5.0.0. Unless otherwise stated, all fixed cell data presented herein are entire Z-series volume projections.

### Electron microscopy

Samples were processed and acquired for transmission electron microscopy as previously described [59].

### Lentiviral knockdown

Wasp, Diaph2 and scrambled shRNA plasmids (a pool of three shRNA plasmids per target) were obtained from Santa Cruz (Santa Cruz, CA). Lentivirus was produced as described above for Lifact-mCherry lentiparticles. For knockdown in the U937 cell line, cells were infected with respective shRNA containing lentivirus at a MOI of 10. Five days post infection, transduced cells were selected by the addition of 2 mg.ml^−1^ puromycin and subsequently passaged in this selective media for 2 weeks. Knockdown at the protein level was verified by western blotting of U937 lysates using the Wasp mAb clone D1 (Santa Cruz) and goat-polyclonal sera (C-12) raised against the C-terminal of Diaph2 (Santa Cruz). Scrambled shRNA (Santa Cruz) control transduced cells were used as the control for shRNA knockdown studies. For knockdown of Diaph2 in primary DCs, we utilized the TRIPZ lentiviral vector (Open Biosystems, Lafayette, CO) that encodes the shRNA sequences in the context of the mir30 scaffold. For DC Transduction, immature DC were transduced with TRIPZ shRNA at a MOI of 1 and cells were subsequently cultured with 1 mg/ml doxycycline to drive TET based shRNA expression. TurboRFP is also under the control of TET in the TRIPZ vector and was used to identify cells that were expressing shRNA.

### Quantitative Cell-Cell transfer assay

To ensure Nef deleted and WT HIV-AD8^ENV^ seeded an equivalent proportion of donor cells, virus was pseudotyped with VSVg envelope. Briefly, VSVg viral stocks were produced by co-transfecting HIV plasmids with the pHEF-VSVG (courtesy of Dr Lung-Ji Chang, through the AIDsreagent respository) at a molar ratio of 1∶1. To further ensure equivalent proportions of infectivity in HIV^NEF^ versus HIV^WT^ in donor cells, their frequency was enumerated by flow cytometry four days post infection by fixation and permeabilization using Fix/Perm and Perm buffers (Becton Dickinson) as described by the manufacturer, in conjunction with the KC57-RD1 (Beckman Coulter, Miami, FL) anti-HIV p24 mAb staining. Following enumeration of the HIV p24 high population in infected DC or CD4 T cells, cells were further normalized using uninfected DC or CD4 T cells from the same donor. Given VSVg pseudotyped stocks were equipotent in viral titers, the latter normalization to 5% was similar between HIV^NEF-ve^and HIV^WT^. The universal recipient for the transfer assay utilized 2×10^5^ activated pure autologous CD4 T cells per well of a tissue culture treated “U” bottom 96 well plate, per 200 ml of RPMI-1640 supplemented with 10% fetal calf serum. After normalization of donor cells, 5×10^4^ cells.50 ml^−1^ were serially diluted at steps of 1/5 dilutions and then added to recipients to give a final co-culture volume of 250 ml. Four days post-co-culture, cells were resolved by CD209-APC (clone DCN46, Becton Dickinson, Franklin Lakes, NJ) staining for DC donor cells, CD3 Alexa Fluor 488 (clone UCHT1, Becton Dickinson) for CD4 T cell recipients and HIV p24 (KC57-RD1 clone, Beckman Coulter, Miami, FL) for enumeration of recipient CD4 T cell infectivity via flow cytometry using a 4 colour FACSCalibur (Becton Dickinson). For each co-culture condition, the sample was acquired in its entirety and represented greater than 30,000 events in the CD3 recipient population in flow cytometric analyses.

For U937 transfer assays, indicated cells were infected at an MOI of 0.1 of HIV and cultured for 48 hours. 48 hours post infection, cells were enumerated as above for DC and subsequently normalized using uninfected control of Diaph2 shRNA transduced U937. After washing and normalization, serial dilutions of 5000 cells were co-cultured with the 20,000 Jurkat indicator cells - JLTRG-R5 (Courtesy of Dr. Olaf Kutsch via the NIH AIDSreagent repository). Four days post infection, infection was enumerated by fluorescence microscopy, as opposed to flow cytometry, as the latter resulted in the loss of detection of GFP positive CD4 -T cell syncytia.

### Statistical analyses

Prior to parametric or non-parametric statistical tests, data herein was tested for normal distribution with the aid of Origin software (Shapiro-Wilk test) (Originlab corporation, Northhampton, MA). Results of experiments have been graphed to exhibit the arithmetic means and standard deviations as indicated in figure legends. When two groups were compared, the probabilities of differences were evaluated by using the nonparametric Mann-Whitney test or, in the case of paired samples, the Wilcoxon matched pairs test. Exact *p* values are stated, unless highly significant different where they are listed as *p*<0.0001. Given the data presented herein utilizes primary cells from different donors, we initially tested each data set for statistical significance between donors to determine the possibility of donor specific outliers. In the latter case where multiple donors are pooled for overall means we list each observations as (*n* = (D = )) where *n* is equally drawn events from “D” number of donors. Random trajectory analysis was carried out by acquiring XY co-ordinates of trajectories after particle tracking using the MtrackJ plugin in ImageJ. Submission of time and XY co-ordinates as a ASCII text file to the website https://weeman.inf.ethz.ch/hurst_estimator/, allows calculation of Hurst Exponents, through previously described algorithms designed by Sbalzerini et al [28] at the Mosaic Group.

## Supporting Information

Figure S1 **Characterising tagged HIV.** **A.** Immature DCs are infected with viral supernatants described in Fig. 1D. In the left panel the psPAX2 rescued HIV iGFP is used to infect immature DCs, whereas in the middle panel the viral stock has been generated using a transfection containing limiting psPAX2 (at a ratio of 8 HIV iGFP plasmid copies to 1 copy of psPAX2). In the far right panel an infection with HIV iGFP is presented alone (non-rescue). All transfections and subsequent infections are outlined as per the legend to Fig. 1D & E and within the materials and methods. Briefly DCs are infected for 4 days with the noted viral preparations, after which cells are fixed, permeabilised and stained for HIV p24 using the KC57-RD1 antibody as described in the materials and methods. HIV p24 staining is presented here on the Y axis with total eGFP expression on the X-axis. Gating is presented herein with the HIV p24 and eGFP postive populations with frequencies presented in the upper right corners. Note all p24 positive cells are also eGFP positive. **B.** Viral spread from infected DCs to autologous CD4 T cells using tagged HIV (HIV iGFP & HIV T) compared to HIV wild type (WT). Immature DCs were infected and normalized as per Fig. 7. The equivalent of 5000 infected DCs were co-cultured with 200,000 activated autologous CD4 T cells for a period of 3 days and then harvested for flow cytometry analysis as outlined in Fig. 7 & materials and methods. The frequency of CD4 T cell infections are presented as gated p24 high populations with frequncies in the upper right corner of gates. The type of virus used is indicated in dot plots at the lower right corner. In the right histogram, flow cytometry data is summarized for the 4 viruses used in this DC- T cell co-culture. Standard deviations are derived from triplicate co-cultures and significant differences are presented as *p* value above the histogram. Data from A and B is representative of *n* = 3 independent donors.
(TIF)

Figure S2 **The role of cellular proteins in VF formation.** **A–D.** Filopodial antigens (red), **A.** Arp2, phosphotyrosine **B.** (pTyr), **C.** WASP and **D.** Cortactin are annotated in volume projected images. HIV iGFP and F-actin staining is outlined in Fig. 1. Yellow arrows highlight particle co-localisation with antigen, whilst white arrows highlight their continuity along the filopodia. All scale bars are at 5 µm. Images are representative of *n* = 7 independent donors. **E.** Verifying protein knockdown in stable U937 cell lines. In the left panels, Wasp and Diaph2 proteins are detected on western blots using the Wasp D1 mAb clone and goat polyclonal sera (C-12) to Diaph2. Scrambled shRNA controls are presented (srshRNA) along side as controls. Lysate controls are presented in the far right panel as western blots probed with anti-Gapdh mAb clone 1D4. **F.** Lack of shRNA knockdown of Diaph2 in primary DC using the same pool of shRNA in E. **G.** Cell free viral production in Diaph2 depleted cells from E. Briefly Diaph2 depleted and untreated U937 cells were infected with VSVg pseudotypes HIV WT and 2 days post infection normalized for percentage infectivity and harvested for cell free virus as outlined in materials and methods. Viral supernatant was analyzed using the TZM.bl HIV indicator cell line as outlined in the legend to Fig. 1. Average absolute b-galactosidase positive cells per well of a 96 well plate are presented with the standard deviations derived from the assay in triplicate. Data is representative of *n* = 3 independent experiments. **H.** Transfer of HIV from infected DCs to autologous CD4 T cells results in productive infection. Dendritic cells were infected and co-cultured with CD4 T cells as outlined in Fig. 7. In the lower panel, 10 µM of AZT is included in the DC- T cell co-culture. Gates in purple are indicative of the productively infected CD4 T cell population.
(TIF)

Video S1 **Untethered DC VF engage in fast Arc trajectories.** Infected DCs with HIV-iGFP were co-cultured with autologous CD4 T cells as outlined in Fig. 1. Frames were acquired at 3 frames/second with DIC and eGFP channels overlaid. Scale bar is presented in the bottom left corner and is in µm. Time is presented after T as hours∶minutes∶Seconds∶Milliseconds. Video is representative of *n* = 20 individual donors.
(QT) Click here for additional data file.

Video S2 **Short filopodia expressed on uninfected DCs.** Uninfected DCs were imaged as outlined in Video S1, with only DIC acquired. Note short filopodia expression opposing the lammelipodia (left of cell). Scale bar is presented in the bottom left corner and is in µm. Duration of Video is 1 minute. Video is representative of *n* = 10 individual donors.
(M4V)

Video S3 **Filopodial triggering on DC during CD4 T cell co-culture.** Uninfected DCs were co-cultured with autologous CD4 T cells as outlined in Fig. 1. Herein a DC expressing similar filopodia to VF is engaging a CD4 T cells presented on the left of the frame. Scale bar is presented in the bottom left corner and is in µm. Duration of Video is 1 minute. Video is representative of *n* = 10 individual donors.
(M4V)

Video S4 **VF expressed on the infected U937 cell line.** The U937 cell line was infected with HIV-iGFP and imaged as outlined in Video S1. Scale bar is presented in the bottom left corner and is in µm. Duration of Video is 1 minute.
(M4V)

Video S5 **Repetitive Arc scanning of HIV VF terminates with CD4 T cell contact.** Infected DCs with HIV-iGFP were co-cultured with autologous CD4 T cells as outlined in Fig. 1. Frames were acquired at 3 frames/second with DIC and eGFP channels overlaid. Scale bar is presented in the bottom left corner is 7 µm. Video is representative of in excess of 120 video files from 6 separate donors. Time, T = is outlined as in Video S1.
(QT) Click here for additional data file.

Video S6 **Fast VF arc velocities, slow to Scan velocities when in contact with CD4 T cells.** VF tips scanning over contacting CD4 T cells. Video is where stills from Fig. 2F are derived and demonstrates the slowing of VF velocities as they scan over the CD4 T cell plasma membrane. Scale bar to the bottom left of the video is 8 µm. Time, T = is outlined in Video S1.
(QT) Click here for additional data file.

Video S7 **Multiple and repetitive HIV VF contacts with CD4 T cells.** Video acquisition and presentation is outlined as per Video S1. Infected DC in the upper field of view maintains multiple filopodial contacts with CD4 T cell presented at the bottom of the field. Scale bar is presented in the bottom left corner and is in µm. Time, T = is outlined in Video S1.
(QT) Click here for additional data file.

Video S8 **Multiple HIV VF contacts leading to CD4 tethering and repositioning.** Video acquisition and presentation is outlined as per Video S1. Infected DC in this field of view commences multiple filopodial contacts with a CD4 T cell in the upper right of the field of view. Contact by multiple filopodia in this instance leads to significant re-positioning of the tethered CD4 T cells closer to the infected DC membrane. Video is where stills of 3D are derived. Scale bar is presented in the bottom left corner and is in µm. Time, T = is outlined in Video S1.
(QT) Click here for additional data file.

Video S9 **High VF activity is associated with CD4 T cell tethering.** Multiple VF contacts result in CD4 T cell tethering. Video is where stills from Fig. 3E. are derived and demonstrate an accumulative VF trajectories for over 4 minutes of an infected DC-CD4 T cell co-culture. HIV-iGFP is presented herein, yet is also representative of HIV^env-ve^-iGFP. Duration of Video is 4 minutes 41 seconds.
(MP4)

Video S10 **Tethering and movement of CD4 T cells engaging multiple VF.** Highlighted CD4 T cell movements over the same 3-minute co-culture presence in Video S8. Video is where stills from Fig. 3E are derived and demonstrate the presence of high filopodial activity with physical tethering and movement of CD4 T cells. CD4 T cell movements are highlighted by trajectories, with neighboring CD4 T cells that are untethered as an example of non-tethered CD4 T cell movements. Duration of Video is as per Video S9.
(M4V)

Video S11 **The PTAP (late domain) of HIV Gag is dispensible for VF formation.** To synchronize immature virions at the plasma membrane of infected cells, we replaced the PTAP motif of the p6 domain in HIV Gag to LIRL. Cell free infectious HIV iGFP lacking the PTAP domain was possible, as HIV iGFP virus is rescued at the protein level using WT Gag and Gag-Pol as outlined in Fig. 1. Thus this approach provides PTAP-Tsg101 recruitment for the generation of infectious cell free virus. Scale bar is 6 µm and time is presented at the bottom left hand corner as per VideoS1. Video is representative of 4 independent infections and over 200 video acquisitions.
(M4V)

Video S12 **Diaph2 stable knockdown of Diaph2 disrupts VF length and trajectories.** The U937 cell line was transduced with lentiviral vectors encoding shRNA against Diaph2 and 3 days post transduction cell were selected with puromycin. After 2 weeks, puromycin resistant cells were infected with HIV iGFP and imaged 2 days post infection. Scale bar is 6 µm and time is presented at the bottom left hand corner as per VideoS1. Video is representative of 50 movies derived from 4 independent infections with HIV iGFP.
(QT) Click here for additional data file.

Video S13 **Diaph2 knockdown in DCs.** Primary DCs were transduced with the TRIPZ shRNAmir against Diaph2, and then subsequently infected with HIV iGFP. In the upper right corner, a smaller video presents cells transduced with shRNAmir, as this vector expresses tRFP (red). In the main video, a shRNAmir transduced DC expressing short VF is presented in the left bottom corner versus an untransduced infected DC in the upper right corner expressing numerous long VF. Scale bar is presented in the bottom left corner and is 10 µm. Time, T = is outlined in Video S1.
(M4V)

Video S14 **Expression of HIV gag alone leads to the formation of VF.** TLC cells that stably express LifeAct-mCherry (mCherry signal presented here in white to image F-actin), were transfected with TI3, a plasmid which encodes HIV Gag-iGFP equivalent to that in HIV iGFP (in green). To aid expression, psPAX2 was co-transfected, to supply WT Gag and rev *in trans*. To image viral filopodia, eGFP positive cells with filopodial activity were selected. Image acquisition, which the exception of acquiring the mCherry signal, was identical to that outlined for primary DC. The movie presented is representative of 20 movies derived from two independent transfections. Scale bar is presented in the left bottom corner at 15 µm. Duration of Video is 1 minute.
(M4V)

Video S15 **VF contacts mature into enveloping CD4 T cell contacts.** HIV-iGFP infected DC expressing VF were imaged after tethering to a CD4 T cell Upper left in field of view) Video is where stills from Fig. 6A. are derived Frames were acquired at 1 frame per 2 minutes with DIC and eGFP channels overlaid. Duration for this movie is 2 hours. Scale bar is at 10 µm. Data is representative of 3 independent donors.
(M4V)
