# Supplementary figures and images for: Mobilization of HIV Spread by Diaphanous 2 Dependent Filopodia in Infected Dendritic Cells
Source: PLoS Pathog. 2012 Jun 7;8(6):e1002762. doi: 10.1371/journal.ppat.1002762 (PMC3369929; doi:10.1371/journal.ppat.1002762)

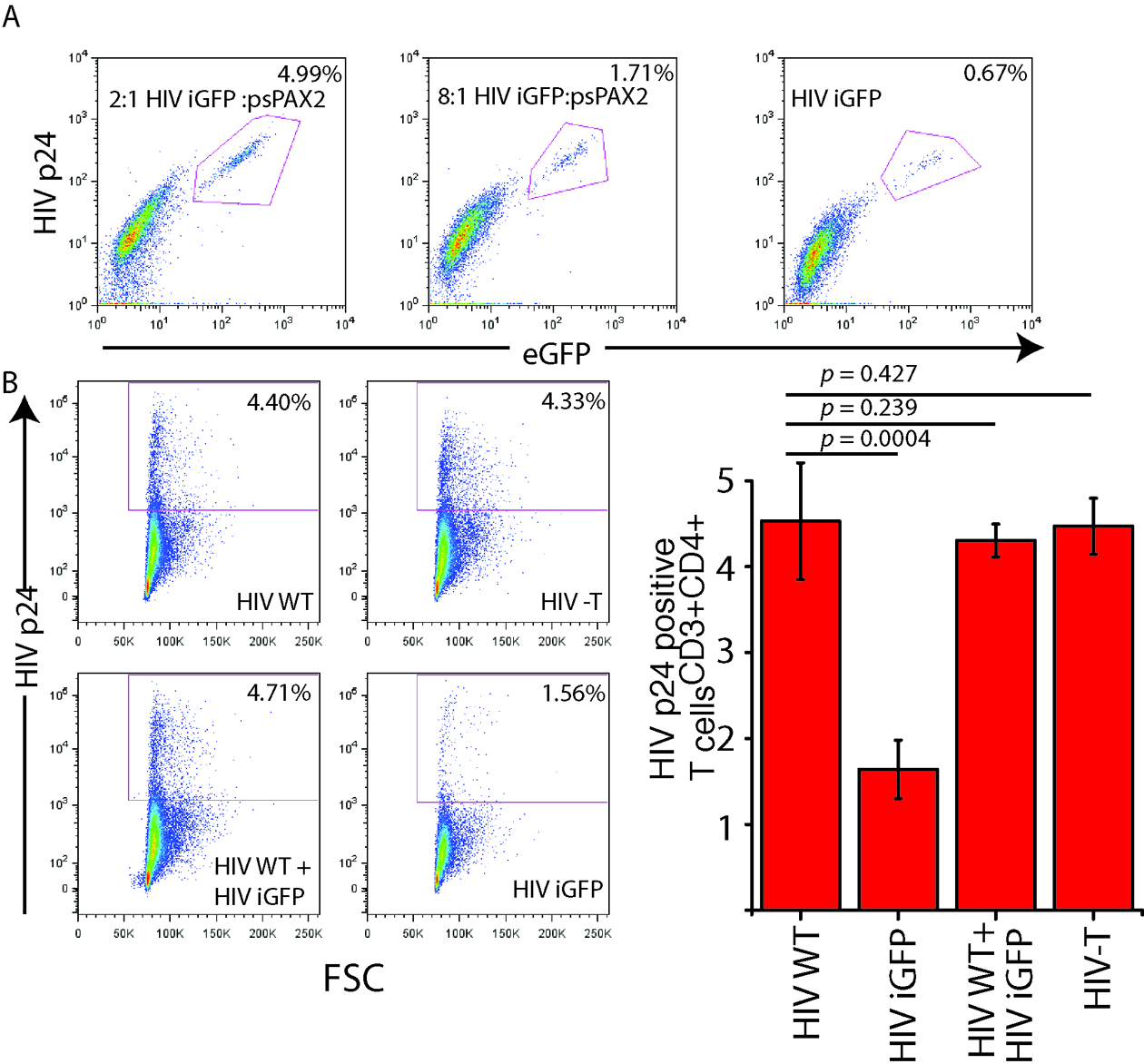

Supplement: Figure S1 — Characterising tagged HIV. A. Immature DCs are infected with viral supernatants described in Fig. 1D. In the left panel the psPAX2 rescued HIV iGFP is used to infect immature DCs, whereas in the middle panel the viral stock has been generated using a transfection containing limiting psPAX2 (at a ratio of 8 HIV iGFP plasmid copies to 1 copy of psPAX2). In the far right panel an infection with HIV iGFP is presented alone (non-rescue). All transfections and subsequent infections are outlined as per the legend to Fig. 1D & E and within the materials and methods. Briefly DCs are infected for 4 days with the noted viral preparations, after which cells are fixed, permeabilised and stained for HIV p24 using the KC57-RD1 antibody as described in the materials and methods. HIV p24 staining is presented here on the Y axis with total eGFP expression on the X-axis. Gating is presented herein with the HIV p24 and eGFP postive populations with frequencies presented in the upper right corners. Note all p24 positive cells are also eGFP positive. B. Viral spread from infected DCs to autologous CD4 T cells using tagged HIV (HIV iGFP & HIV T) compared to HIV wild type (WT). Immature DCs were infected and normalized as per Fig. 7. The equivalent of 5000 infected DCs were co-cultured with 200,000 activated autologous CD4 T cells for a period of 3 days and then harvested for flow cytometry analysis as outlined in Fig. 7 & materials and methods. The frequency of CD4 T cell infections are presented as gated p24 high populations with frequncies in the upper right corner of gates. The type of virus used is indicated in dot plots at the lower right corner. In the right histogram, flow cytometry data is summarized for the 4 viruses used in this DC- T cell co-culture. Standard deviations are derived from triplicate co-cultures and significant differences are presented as p value above the histogram. Data from A and B is representative of n = 3 independent donors. (TIF) [file ppat.1002762.s001.tif]

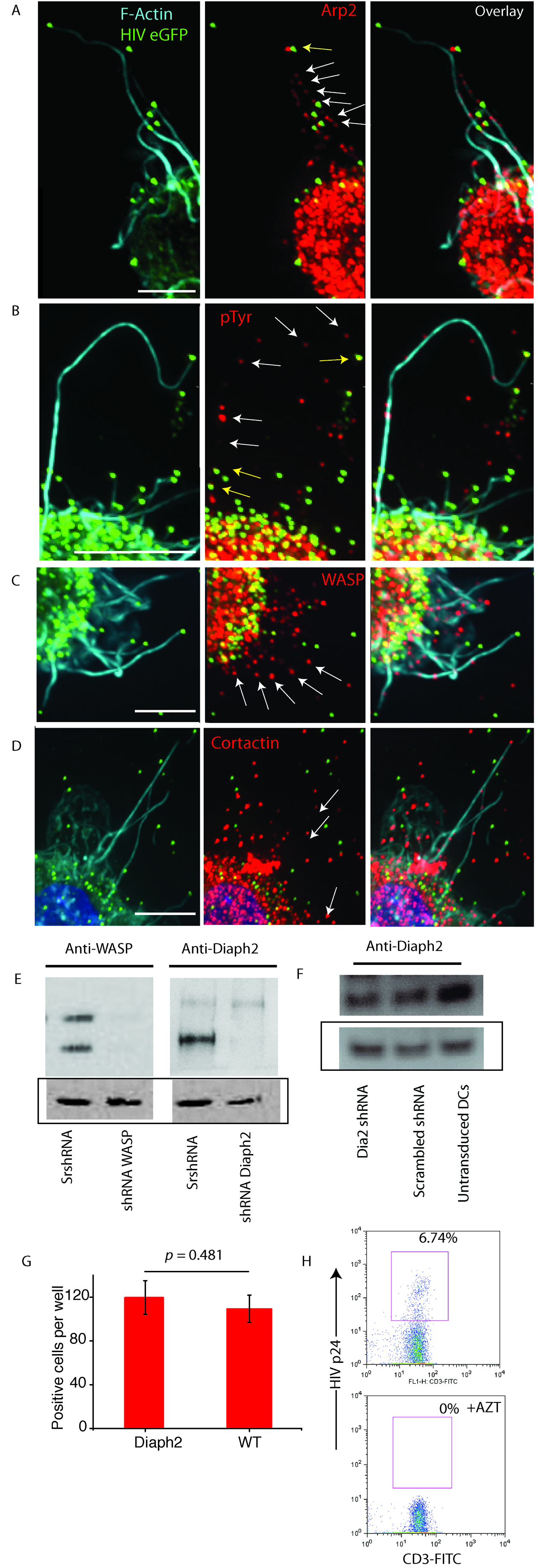

Supplement: Figure S2 — The role of cellular proteins in VF formation. A–D. Filopodial antigens (red), A. Arp2, phosphotyrosine B. (pTyr), C. WASP and D. Cortactin are annotated in volume projected images. HIV iGFP and F-actin staining is outlined in Fig. 1. Yellow arrows highlight particle co-localisation with antigen, whilst white arrows highlight their continuity along the filopodia. All scale bars are at 5 µm. Images are representative of n = 7 independent donors. E. Verifying protein knockdown in stable U937 cell lines. In the left panels, Wasp and Diaph2 proteins are detected on western blots using the Wasp D1 mAb clone and goat polyclonal sera (C-12) to Diaph2. Scrambled shRNA controls are presented (srshRNA) along side as controls. Lysate controls are presented in the far right panel as western blots probed with anti-Gapdh mAb clone 1D4. F. Lack of shRNA knockdown of Diaph2 in primary DC using the same pool of shRNA in E. G. Cell free viral production in Diaph2 depleted cells from E. Briefly Diaph2 depleted and untreated U937 cells were infected with VSVg pseudotypes HIV WT and 2 days post infection normalized for percentage infectivity and harvested for cell free virus as outlined in materials and methods. Viral supernatant was analyzed using the TZM.bl HIV indicator cell line as outlined in the legend to Fig. 1. Average absolute b-galactosidase positive cells per well of a 96 well plate are presented with the standard deviations derived from the assay in triplicate. Data is representative of n = 3 independent experiments. H. Transfer of HIV from infected DCs to autologous CD4 T cells results in productive infection. Dendritic cells were infected and co-cultured with CD4 T cells as outlined in Fig. 7. In the lower panel, 10 µM of AZT is included in the DC- T cell co-culture. Gates in purple are indicative of the productively infected CD4 T cell population. (TIF) [file ppat.1002762.s002.tif]
